# Forskolin-mediated cAMP activation upregulates TNF-α expression despite NF-κB downregulation in LPS-treated Schwann cells

**DOI:** 10.1371/journal.pone.0302223

**Published:** 2024-04-16

**Authors:** Caitlyn Henry, Mackenzie Wilcox, Angela L. Asirvatham

**Affiliations:** Department of Biology, Misericordia University, Dallas, PA, United States of America; Northwest University, UNITED STATES

## Abstract

Although Schwann cells have been found to play a key role in inflammation and repair following nerve injury, the exact pathway is still unknown. To explore the mechanism by which Schwann cells exert their effects in the neuron microenvironment, we investigated two main inflammatory pathways: the NF-κB and cAMP pathways, and their downstream signaling molecules. In this study, lipopolysaccharide (LPS), a bacterial endotoxin, was used to activate the NF-κB pathway, and forskolin, a plant extract, was used to activate the cAMP pathway. The rat RT4-D6P2T Schwann cell line was treated with 0.1, 1, or 10 μg/mL of LPS, with or without 2 μM of forskolin, for 1, 3, 12, and 24 hours to determine the effects of elevated cAMP levels on LPS-treated cell viability. To investigate the effects of elevated cAMP levels on the expression of downstream signaling effector proteins, specifically NF-κB, TNF-α, AKAP95, and cyclin D3, as well as TNF-α secretion, RT4-D6P2T cells were incubated in the various treatment combinations for a 3-hour time period. Overall, results from the CellTiter-Glo viability assay revealed that forskolin increased viability in cells treated with smaller doses of LPS for 1 and 24 hours. For all time points, 10 μg/mL of LPS noticeably reduced viability regardless of forskolin treatment. Results from the Western blot analysis revealed that, at 10 μg/mL of LPS, forskolin upregulated the expression of TNF-α despite a downregulation of NF-κB, which was also accompanied by a decrease in TNF-α secretion. These results provide evidence that cAMP might regulate TNF-α expression through alternate pathways. Furthermore, although cAMP activation altered AKAP95 and cyclin D3 expression at different doses of LPS, there does not appear to be an association between the expression of AKAP95 or cyclin D3 and the expression of TNF-α. Exploring the possible interactions between cAMP, NF-κB, and other key inflammatory signaling pathways might reveal a potential therapeutic target for the treatment of nerve injury and inflammation.

## Introduction

Schwann cells are the principal glial cells of the peripheral nervous system. Their main function is to produce the myelin sheath that insulates neurons and promotes the rapid conduction of electrical impulses throughout the body. In addition to their basic functions, Schwann cells demonstrate the ability to mediate axonal regeneration following nerve injury and inflammation.

During nerve injury, the myelin sheath is damaged, initiating an inflammatory response. Schwann cells secrete macrophage inflammatory protein-1α (MIP-1α) and other cytokines that stimulate immune cells, such as macrophages, to travel to the site of the nerve lesion and facilitate the clearance of myelin debris [1]. Once the debris is cleared, the neuron secretes neuronal mitogens that activate the cAMP pathway to promote Schwann cell proliferation so the myelin sheath can be replenished [2,3]. Considering the inability of neurons to regenerate themselves, an understanding of the signaling pathways that mediate axonal repair by glial cells might help to find a potential therapeutic target for the treatment of nerve injury and inflammation.

This paper investigates two key signaling pathways that are known to regulate inflammation: the nuclear factor kappa B (NF-κB) and cAMP pathways. NF-κB is a universal transcription factor involved in innate and adaptive immunity, cell survival, growth, and development [4,5]. It can be found in many different dimeric forms, with the most common form being the p50/p65 heterodimer [5]. Under non-inflammatory conditions, the p50/p65 heterodimer is sequestered in the cytoplasm by inhibitor of kappa B (IκB). Once the cell receives an inflammatory signal, a series of signaling events activates IκB kinase (IKK), which phosphorylates IκB and tags it for degradation [5]. The degradation of IκB liberates the p50/p65 heterodimer, allowing it to translocate into the nucleus and bind to genes responsible for the production of various pro- and anti-inflammatory cytokines [5]. Tumor necrosis factor alpha (TNF-α) is one of the cytokines produced by Schwann cells [6] via the NF-κB pathway [5,7,8] and has also been found to regulate NF-κB [8]. Thus, TNF-α is an important protein to consider when studying the role of the NF-κB signaling pathway in Schwann cells during nerve injury and inflammation.

The cAMP pathway is a universal signaling pathway involved in various biological processes, including cell proliferation, differentiation, and apoptosis [9]. A recent study demonstrated cAMP’s ability to promote Schwann cell proliferation through the upregulation of A-kinase anchoring protein 95 (AKAP95) and cyclin D3 [9]. AKAP95 is a scaffolding protein that coordinates downstream signaling events in the nucleus, and cyclin D3 is a cell cycle regulatory protein [9]. The cAMP pathway is also known to be anti-inflammatory in addition to its proliferative effects. For example, cAMP has been found to suppress TNF-α mRNA expression via PKA and AKAP95 in LPS-treated macrophages [10]. Due to the abilities of AKAP95 and cyclin D3 to promote Schwann cell proliferation, as well as the potential role of AKAP95 in mediating inflammation, it is important to consider these proteins when studying the role of the cAMP signaling pathway in Schwann cells during nerve injury and inflammation.

*In vitro*, the NF-κB pathway can be activated by treating cells with lipopolysaccharide (LPS), a cell wall immunostimulatory component of Gram-negative bacteria [4], and the cAMP pathway can be activated by treating cells with forskolin, a root extract from the Indian plant *Coleus forskohlii* [9]. Although it is well known that the NF-κB and cAMP pathways are both involved in inflammation, additional research regarding the effects of forskolin-mediated cAMP activation on LPS-treated Schwann cells in terms of cell viability, protein expression, and TNF-α secretion, will further our understanding of the potential interactions between these two signaling pathways to mediate axonal repair. We hypothesized that LPS-treated cells stimulated with forskolin would have more viability than cells without forskolin. We also hypothesized that LPS-treated cells with forskolin would have less NF-κB and TNF-α expression, and in turn less TNF-α secretion, as well as more AKAP95 and cyclin D3 expression, compared to the control. Our results suggest that activating cAMP in LPS-treated Schwann cells might alter TNF-α expression through signaling pathways that may or may not involve NF-κB. Furthermore, we found that AKAP95 and cyclin D3 expression do not necessarily correlate with TNF-α expression in LPS-treated Schwann cells.

## Results

### The effects of forskolin on the viability of LPS-treated RT4-D6P2T cells

A CellTiter-Glo viability assay was performed to assess the effects of forskolin on RT4-D6P2T Schwann cells treated with various doses of LPS. Relative luminescence units are displayed as a mean percent control (N_2_) ± standard error of the mean (SEM). Results from three independent experiments were analyzed using one-way ANOVA and tested with Tukey’s and least significant difference (LSD) post-hoc analysis (S1 Table).

**Fig 1:**
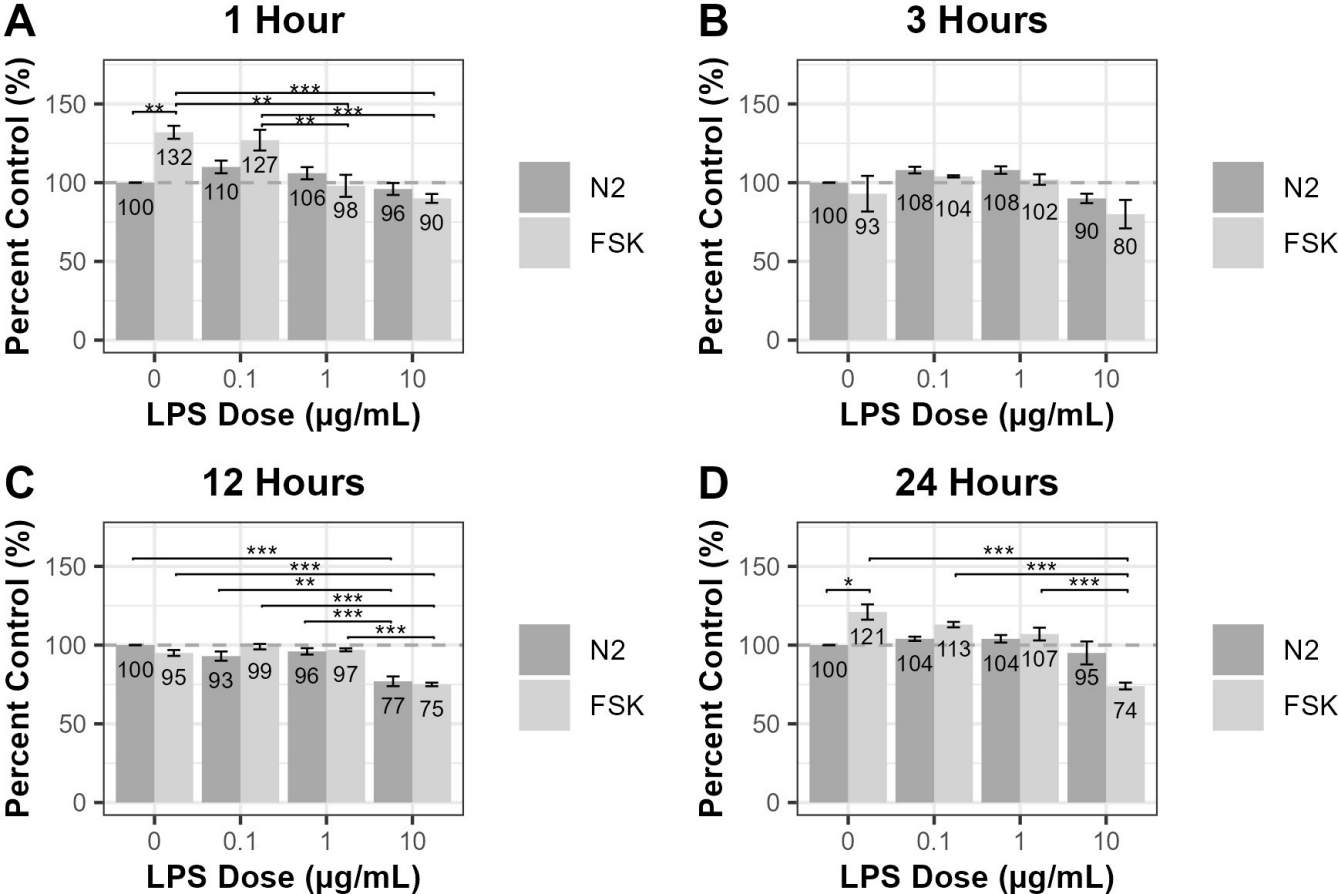
The effects of forskolin on the viability of LPS-treated RT4-D6P2T cells. Using the CellTiter-Glo 2.0 Assay (Promega), the immortalized rat RT4-D6P2T Schwann cell line (ATCC #CRL-2768) was treated with 0.1, 1, or 10 μg/mL of LPS in N_2_ media, with or without 2 μM of forskolin, for (A) 1, (B) 3, (C) 12, and (D) 24 hours. Relative luminescence units were read as an indicator of viability and are displayed as a mean percent control ± SEM. The dotted line indicates a percent control of 100%, with a percent control above 100% representing increased relative luminescence units (more viable cells) and a percent control below 100% representing decreased relative luminescence units (less viable cells) compared to the N_2_ control. Results from all experiments were examined using one-way ANOVA and tested with Tukey’s and LSD post-hoc analysis in R Studio. Mean percent controls with the same number of asterisks (*) are significantly different from each other (*p < 0.05, **p < 0.01, ***p < 0.001) (n = 3).

Results from the RT4-D6P2T assay indicated that for 1-, 3-, and 24-hour cultures, at 0.1 μg/mL and 1 μg/mL of LPS, cell viability either increased or stayed the same, regardless of forskolin treatment (Fig 1). Overall, it appears that a treatment of 10 μg/mL of LPS led to the most notable decrease in cell viability (Fig 1 and S1 Table).

Because cell viability appears to decrease with increasing doses of LPS, a Pearson’s correlation test was performed to determine whether there is an association between LPS dose and mean percent control, regardless of forskolin treatment. Pearson’s correlation test revealed a strong (r < -0.8) or moderately strong (-0.8 < r < -0.6), negative linear relationship between LPS dose and mean percent control of cells cultured in control and forskolin-supplemented media for all time points, with the exception of cells in forskolin-supplemented media for 3 hours and cells in control media for 24 hours (Table 1). All results are statistically significant (*p* < 0.05), with the exception of 24-hour cultures in control media.

**Table 1:** Pearson’s correlation coefficients and *p*-values.

Groups Compared	Pearson’s Coefficient (r)	*p*-value
LPS dose vs. cell viability	-	-
1 hour (N_2_)	-0.6013	0.0386 *
3 hours (N_2_)	-0.8764	1.836 x 10–4 *
12 hours (N_2_)	-0.8347	7.312 x 10–4 *
24 hours (N_2_)	-0.5726	0.0517
1 hour (Fsk)	-0.6969	0.0118 *
3 hours (Fsk)	-0.5812	0.0475 *
12 hours (Fsk)	-0.9603	7.262 x 10–7 *
24 hours (Fsk)	-0.9441	3.918 x 10–6 *
NF-κB expression vs. TNF-α expression	0.6058	0.0368 *
AKAP95 expression vs. TNF-α expression	0.0464	0.8863
Cyclin D3 expression vs. TNF-α expression	0.1104	0.7327

Using R Statistical Software (v4.2.2; R Core Team 2022), the degree of the linear relationship between different variables was determined by performing a series of Pearson’s correlation tests (*p < 0.05).

Additionally, for the 1- and 3-hour time points, cells treated with forskolin had a lower mean percent control than cells cultured in N_2_ media only (control) regardless of LPS dose, except for cells treated without LPS and with 0.1 μg/mL of LPS for 1 hour (Fig 1A and 1B). For the 12-hour time point, cells treated with forskolin and 0.1 or 1 μg/mL of LPS (99.10 ± 1.72% and 97.36 ± 0.95%, respectively) had a greater mean percent control than the N_2_ cultures (92.77 ± 2.93% and 96.14 ± 2.03%, respectively), while cells treated with forskolin and 0 or 10 μg/mL of LPS (95.11 ± 1.86% and 75.29 ± 1.13%, respectively) had a lower mean percent control than the N_2_ cultures (100.00 ± 0.00% and 77.48 ± 3.09%, respectively) (Fig 1C). For the 24-hour time point, cells treated with forskolin and 0, 0.1, or 1 μg/mL of LPS (121.08 ± 4.86%, 113.43 ± 1.72%, and 106.86 ± 4.05%, respectively) had a greater mean percent control than the N_2_ cultures (100.00 ± 0.00%, 103.98 ± 1.34%, and 104.02 ± 2.38%, respectively), while forskolin-supplemented media decreased viability in cells treated with 10 μg/mL of LPS compared to the control (74.19 ± 2.15% and 95.25 ± 7.28%, respectively) (Fig 1D).

The CellTiter-Glo viability assay was repeated in the rat S16 Schwann cell line, and results indicated similar patterns to the RT4-D6P2T cell viability assay. For all time points, 10 μg/mL of LPS decreased cell viability, regardless of forskolin treatment, with the exception of cells incubated in control media for 3-hours (S1 Fig).

### The effects of forskolin on NF-κB and TNF-α expression in LPS-treated RT4-D6P2T cells

Immunoblotting was performed to assess the effects of forskolin on NF-κB and TNF-α expression in RT4-D6P2T cells treated with various doses of LPS for 3 hours. Relative band densities are displayed as mean fold change ± SEM. Results from three independent experiments were examined using one-way ANOVA and tested with Tukey’s and LSD post-hoc analysis (S2 Table).

**Fig 2:**
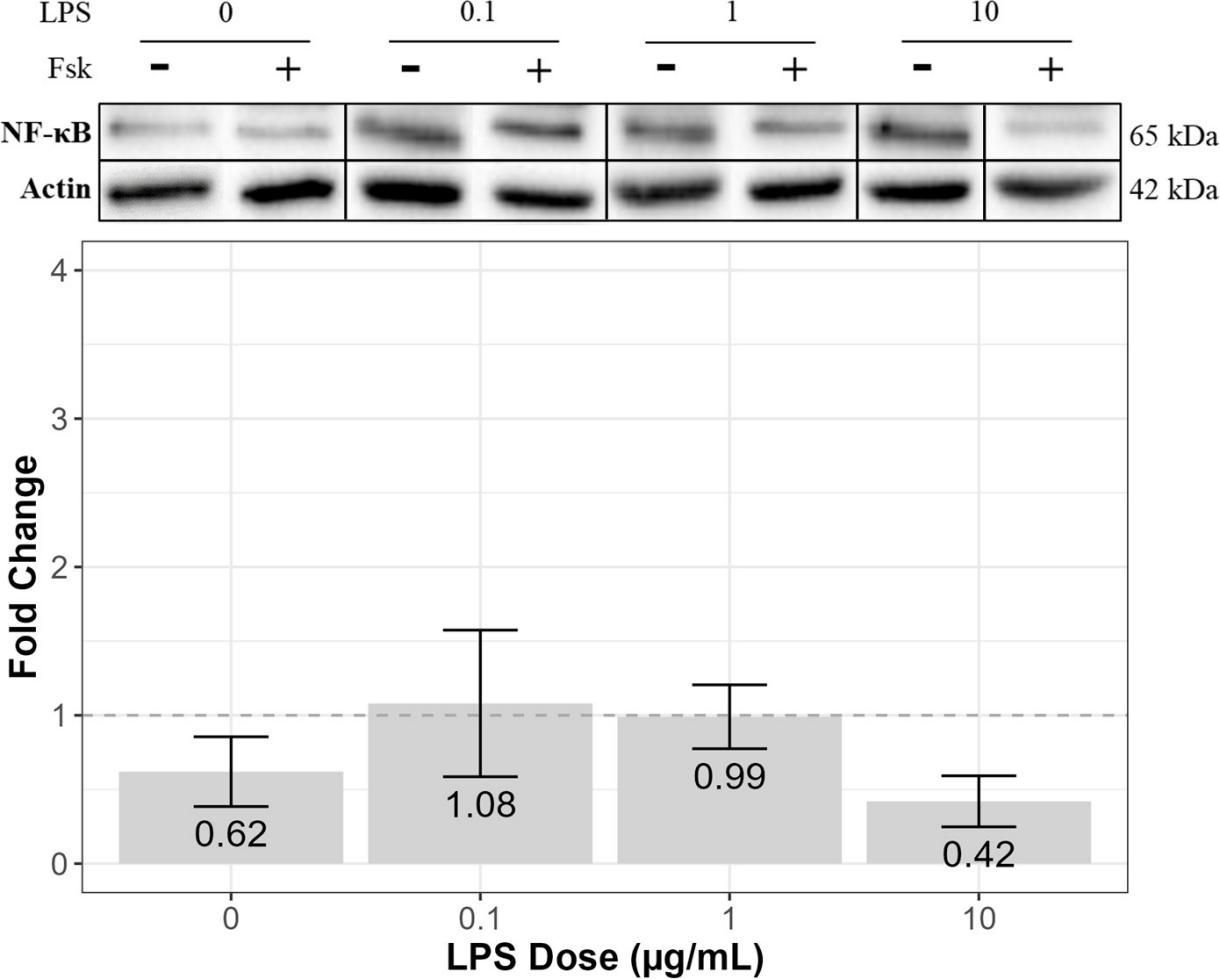
The effects of forskolin on NF-κB expression in LPS-treated RT4-D6P2T cells. The immortalized rat RT4-D6P2T Schwann cell line (ATCC #CRL-2768) was treated with 0.1, 1, or 10 μg/mL of LPS in N_2_ media, with or without 2 μM of forskolin (+/- Fsk), for 3 hours. SDS-PAGE gel electrophoresis and Western blotting were performed using prepared cell lysates. NF-κB expression was visualized using enhanced chemiluminescence reagent and quantified via densitometry analysis using Bio Rad Image software. Actin was used as a loading control, and detected expression levels of actin were used to normalize all Western blots. The above blots are representative blots from three independent experiments that were spliced together, as indicated by the vertical black lines. Relative band densities are displayed as mean fold change (over the N_2_ control [no LPS]) ± SEM. The dotted line indicates a fold change of 1, with a fold change above 1 representing increased protein expression and a fold change below 1 representing decreased protein expression compared to the N_2_ control. Results from three independent experiments were examined using one-way ANOVA and tested with Tukey’s and LSD post-hoc analysis in R Studio. There was no significant difference in mean fold change between the various doses of LPS (F = 0.240, df = 1, *p* = 0.635) (n = 3).

For cells treated with forskolin only (no LPS), NF-κB expression was downregulated compared to the control (0.62 ± 0.24) (Fig 2). Forskolin treatment also downregulated NF-κB expression in cells treated with 10 μg/mL of LPS compared to the control (0.42 ± 0.17) (Fig 2). However, forskolin treatment did not alter NF-κB expression in cells treated with 0.1 and 1 μg/mL of LPS compared to the control (1.08 ± 0.49 and 0.99 ± 0.22, respectively) (Fig 2).

**Fig 3:**
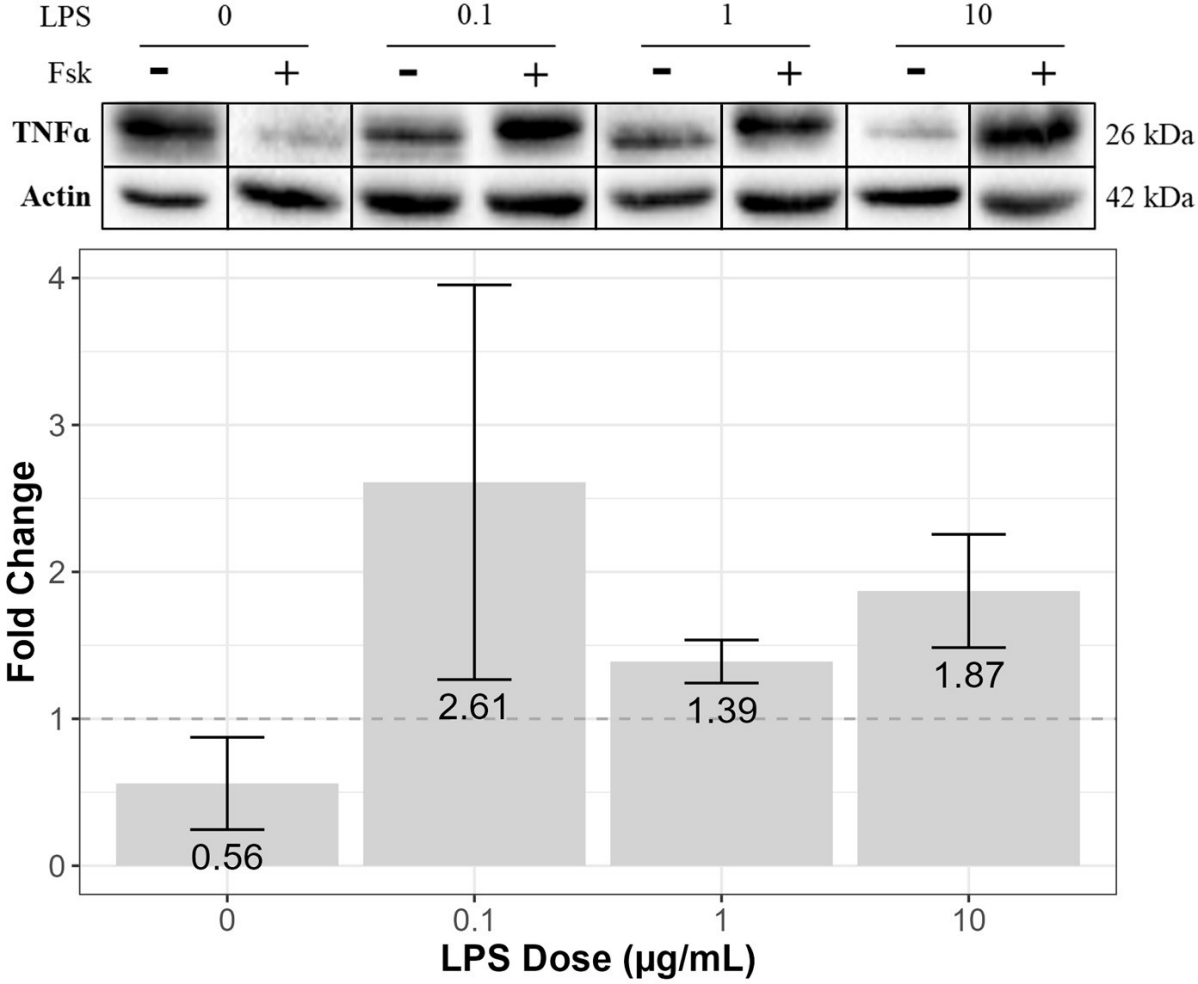
The effects of forskolin on TNF-α expression in LPS-treated RT4-D6P2T cells. The immortalized rat RT4-D6P2T Schwann cell line (ATCC #CRL-2768) was treated with 0.1, 1, or 10 μg/mL of LPS in N_2_ media, with or without 2 μM of forskolin (+/- Fsk), for 3 hours. SDS-PAGE gel electrophoresis and Western blotting were performed using prepared cell lysates. TNF-α expression was visualized using enhanced chemiluminescence reagent and quantified via densitometry analysis using Bio Rad Image software. Actin was used as a loading control, and detected expression levels of actin were used to normalize all Western blots. The above blots are representative blots from three independent experiments that were spliced together, as indicated by the vertical black lines. Relative band densities are displayed as mean fold change (over the N_2_ control [no LPS]) ± SEM. The dotted line indicates a fold change of 1, with a fold change above 1 representing increased protein expression and a fold change below 1 representing decreased protein expression compared to the N_2_ control. Results from three independent experiments were examined using one-way ANOVA and tested with Tukey’s and LSD post-hoc analysis in R Studio. There was no significant difference in mean fold change between the various doses of LPS (F = 0.613, df = 1, *p* = 0.452) (n = 3).

In terms of TNF-α expression, for cells treated with forskolin only, TNF-α expression was downregulated compared to the control (0.56 ± 0.31) (Fig 3). However, forskolin treatment upregulated TNF-α expression in cells treated with 0.1, 1, and 10 μg/mL of LPS compared to the control (2.61 ± 1.34, 1.39 ± 0.15, and 1.87 ± 0.39, respectively), with forskolin upregulating TNF-α expression the most in cells treated with 0.1 μg/mL of LPS (Fig 3). Furthermore, results from Pearson’s correlation revealed a moderately strong (0.6 < r < 0.8), positive linear relationship between NF-κB expression and TNF-α expression (Table 1).

Overall, at 0.1 μg/mL of LPS with forskolin, expression of NF-κB was unaffected, and TNF-α was upregulated, while cell viability remained unchanged, compared to the control. On the other hand, at 10 μg/mL of LPS with forskolin, expression of NF-κB was downregulated, and TNF-α was still upregulated, while cell viability declined, compared to the control.

### The effects of forskolin on AKAP95 and cyclin D3 expression in LPS-treated RT4-D6P2T cells

Immunoblotting was performed to assess the effects of forskolin on AKAP95 and cyclin D3 expression in RT4-D6P2T cells treated with various doses of LPS for 3 hours. Relative band densities are displayed as mean fold change ± SEM. Results from three independent experiments were examined using one-way ANOVA and tested with Tukey’s and LSD post-hoc analysis (S2 Table).

**Fig 4:**
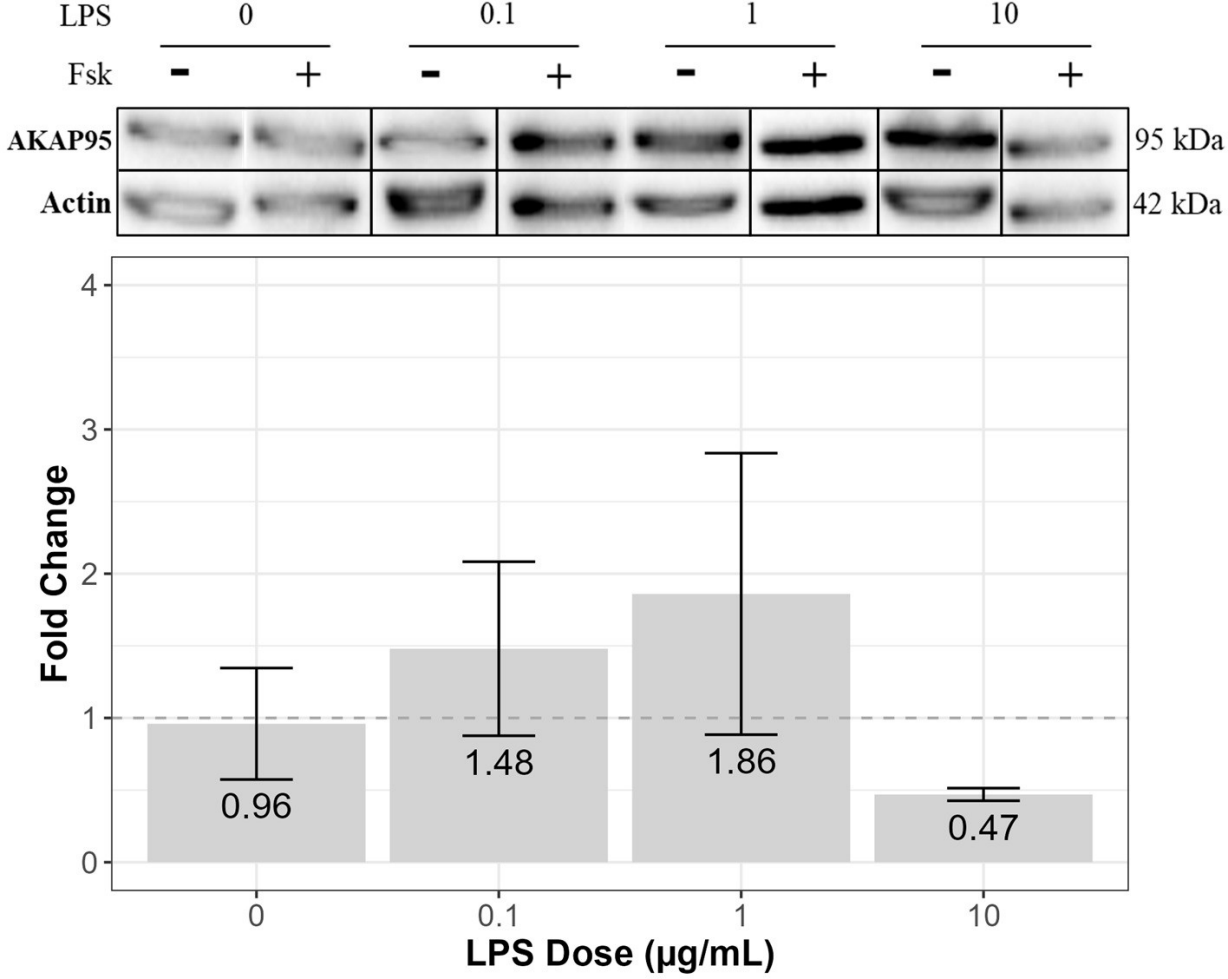
The effects of forskolin on AKAP95 expression in LPS-treated RT4-D6P2T cells. The immortalized rat RT4-D6P2T Schwann cell line (ATCC #CRL-2768) was treated with 0.1, 1, or 10 μg/mL of LPS in N_2_ media, with or without 2 μM of forskolin (+/- Fsk), for 3 hours. SDS-PAGE gel electrophoresis and Western blotting were performed using prepared cell lysates. AKAP95 expression was visualized using enhanced chemiluminescence reagent and quantified via densitometry analysis using Bio Rad Image software. Actin was used as a loading control, and detected expression levels of actin were used to normalize all Western blots. The above blots are representative blots from three independent experiments that were spliced together, as indicated by the vertical black lines. Relative band densities are displayed as mean fold change (over the N_2_ control [no LPS]) ± SEM. The dotted line indicates a fold change of 1, with a fold change above 1 representing increased protein expression and a fold change below 1 representing decreased protein expression compared to the N_2_ control. Results from three independent experiments were examined using one-way ANOVA and tested with Tukey’s and LSD post-hoc analysis in R Studio. There was no significant difference in mean fold change between the various doses of LPS (F = 0.151, df = 1, *p* = 0.706) (n = 3).

For cells treated with N_2_ media only (no LPS), forskolin treatment did not have much of an effect on AKAP95 expression compared to the control (0.96 ± 0.39) (Fig 4). However, forskolin treatment upregulated AKAP95 expression in cells treated with 0.1 and 1 μg/mL of LPS (1.48 ± 0.60 and 1.86 ± 0.98, respectively), while it downregulated AKAP95 expression in cells treated with 10 μg/mL of LPS compared to the control (0.47 ± 0.04) (Fig 4). Furthermore, results from Pearson’s correlation revealed no linear relationship between AKAP95 expression and TNF-α expression (Table 1).

**Fig 5:**
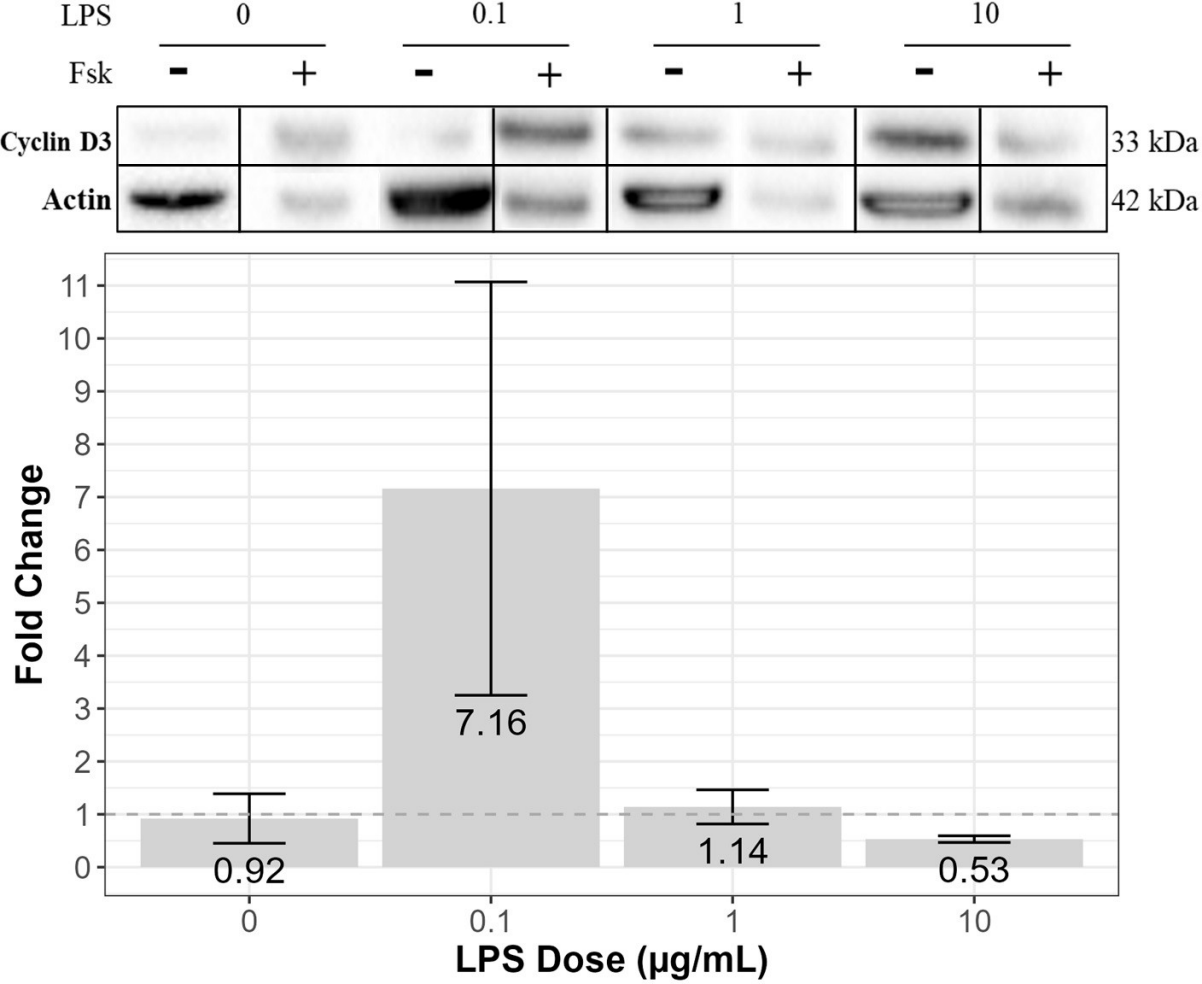
The effects of forskolin on cyclin D3 expression in LPS-treated RT4-D6P2T cells. The immortalized rat RT4-D6P2T Schwann cell line (ATCC #CRL-2768) was treated with 0.1, 1, or 10 μg/mL of LPS in N_2_ media, with or without 2 μM of forskolin (+/- Fsk), for 3 hours. SDS-PAGE gel electrophoresis and Western blotting were performed using prepared cell lysates. Cyclin D3 expression was visualized using enhanced chemiluminescence reagent and quantified via densitometry analysis using Bio Rad Image software. Actin was used as a loading control, and detected expression levels of actin were used to normalize all Western blots. The above blots are representative blots from three independent experiments that were spliced together, as indicated by the vertical black lines. Relative band densities are displayed as mean fold change (over the N_2_ control [no LPS]) ± SEM. The dotted line indicates a fold change of 1, with a fold change above 1 representing increased protein expression and a fold change below 1 representing decreased protein expression compared to the N_2_ control. Results from three independent experiments were examined using one-way ANOVA and tested with Tukey’s and LSD post-hoc analysis in R Studio. There was no significant difference in mean fold change between the various doses of LPS (F = 0.438, df = 1, *p* = 0.523) (n = 3).

In terms of cyclin D3, forskolin treatment did not alter expression in cells treated with N_2_ media only (no LPS) or cells treated with 1 μg/mL of LPS compared to the control (0.92 ± 0.47 and 1.14 ± 0.32, respectively) (Fig 5). However, forskolin upregulated cyclin D3 expression in cells treated with 0.1 μg/mL of LPS and downregulated cyclin D3 expression in cells treated with 10 μg/mL of LPS (7.16 ± 3.91 and 0.53 ± 0.06) (Fig 5). Furthermore, results from Pearson’s correlation revealed no linear relationship between cyclin D3 expression and TNF-α expression (Table 1).

Overall, at 0.1 μg/mL of LPS with forskolin, AKAP95 and cyclin D3 expression were upregulated, while cell viability remained unchanged, compared to the control. On the other hand, at 10 μg/mL of LPS with forskolin, AKAP95 and cyclin D3 expression were downregulated, while cell viability declined, compared to the control.

### The effects of forskolin on TNF-α secretion by LPS-treated RT4-D6P2T cells

**Fig 6:**
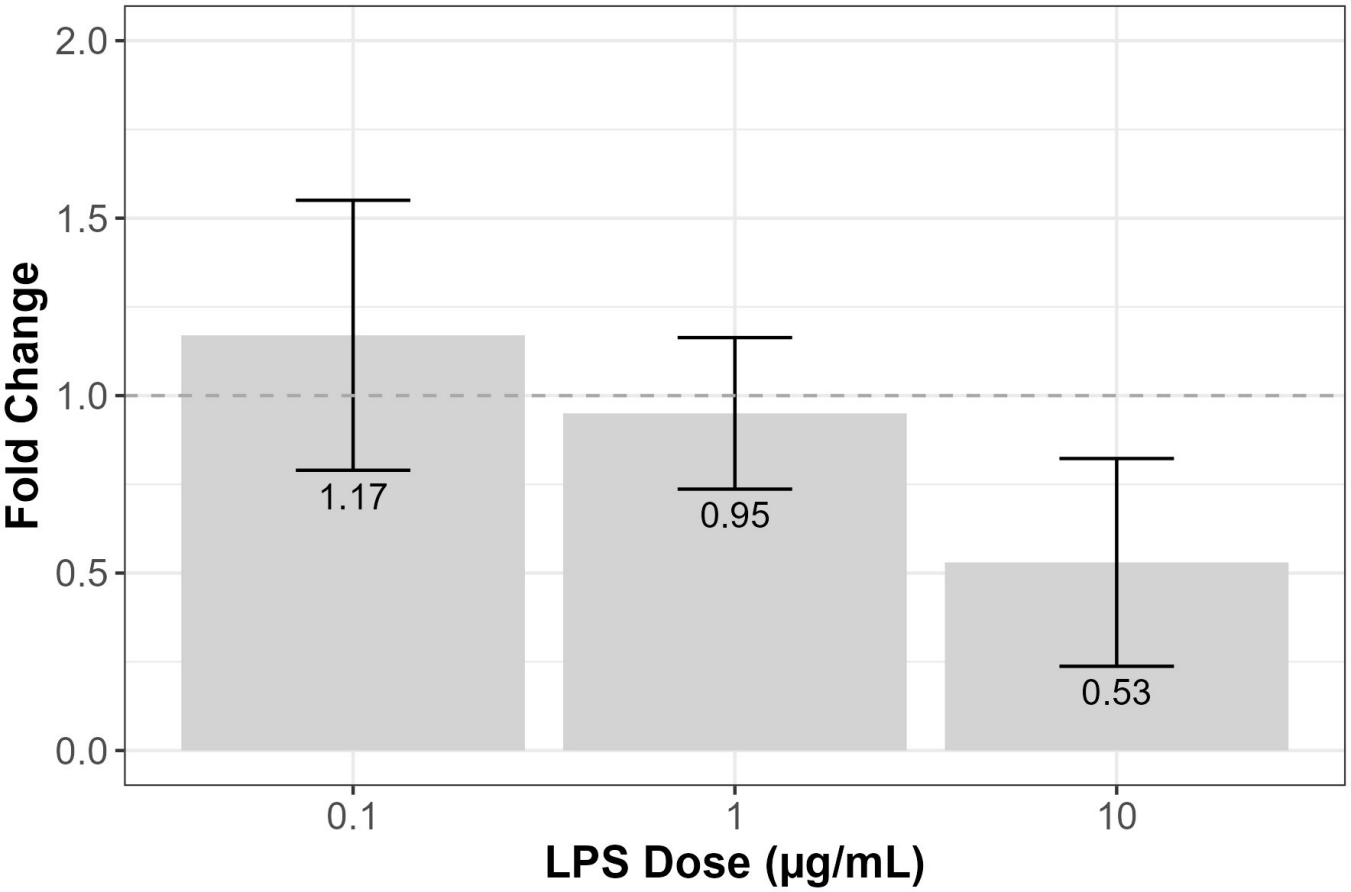
The effects of forskolin on TNF-α secretion by LPS-treated RT4-D6P2T cells. The immortalized rat RT4-D6P2T Schwann cell line (ATCC #CRL-2768) was treated with 0.1, 1, or 10 μg/mL of LPS in N_2_ media, with or without 2 μM of forskolin, for 3 hours. The Invitrogen Rat TNF alpha ELISA kit (RayBiotech) was used to quantify the TNF-α concentration (pg/mL) in media samples, which is displayed as mean fold change ± SEM. The dotted line indicates a fold change of 1, with a fold change above 1 representing increased TNF-α secretion and a fold change below 1 representing decreased TNF-α secretion compared to the N_2_ control. Results from three independent experiments were examined using one-way ANOVA and tested with Tukey’s and LSD post-hoc analysis in R Studio. There was no significant difference in mean fold change between the various doses of LPS (F = 2.437, df = 1, *p* = 0.147) (n = 3).

TNF ELISA was performed to assess the effects of forskolin on TNF-α secretion by RT4-D6P2T cells treated with various doses of LPS for 3 hours. TNF-α secretion is displayed as mean fold change ± SEM. Results from three independent experiments were examined using one-way ANOVA and tested with Tukey’s and LSD post-hoc analysis. For cells treated with 0.1 μg/mL of LPS, forskolin treatment increased TNF-α secretion compared to the control (1.17 ± 0.38) (Fig 6). On the other hand, for cells treated with 1 and 10 μg/mL of LPS, forskolin decreased TNF-α secretion compared to the control (0.95 ± 0.21 and 0.53 ± 0.29, respectively) (Fig 6).

## Discussion

This study depicts the effects of cAMP activation on Schwann cells during LPS-mediated NF-κB activation. Using forskolin to activate the cAMP pathway and different doses of LPS to activate the NF-κB pathway, we demonstrated cAMP’s ability to alter viability, protein expression, and TNF-α secretion, in LPS-treated RT4-D6P2T cells.

Throughout this study, RT4-D6P2T cells were treated with 0.1, 1, or 10 μg/mL of LPS, with or without 2 μM of forskolin. For the cell viability assay, cells were subjected to a 1-, 3-, 12-, or 24-hour incubation time, and for the immunoblotting and TNF ELISA experiments, cells were subjected to a 3-hour incubation time. A 2 μM dose of forskolin was selected based on the results of an unpublished forskolin dose response (S2 Fig). Regarding the LPS doses and incubation times, preliminary studies have shown that in forskolin-treated S16 Schwann cells, viability was highest in cells treated with LPS for 24 hours. However, the transcription time frame for NF-κB is approximately 100 minutes in neuroblastoma cells [11]. Wall et al. found that in macrophages treated with 100 ng/mL (0.1 μg/mL) of LPS, mRNA expression of TNF-α significantly increased within the first hour and leveled off thereafter [10]. Another study by Cheng et al. demonstrated that in primary rat Schwann cells treated with 10 μg/mL of LPS, TNF-α synthesis was highest after a 3-hour incubation period [7]. Therefore, we used time points of 1, 3, 12, and 24 hours in our study to account for the rapid nature of NF-κB transcription and TNF-α production while ensuring sufficient time for maximal Schwann cell proliferation. We used LPS doses of 0.1, 1, and 10 μg/mL in response to the studies conducted by Cheng et al. and Wall et al. [7,10].

According to results from the CellTiter-Glo viability assay, for the 1- and 24-hour time points, cells cultured with or without 0.1 μg/mL of LPS in forskolin-supplemented media experienced an improvement in cell viability. However, for the 3- and 12- hour time points, forskolin did not affect the viability of control cells or cells treated with 0.1 or 1 μg/mL of LPS. On the other hand, 10 μg/mL of LPS impaired cell viability for all time points, with cells cultured in forskolin experiencing a greater reduction in viability compared to control cells. Overall, it appears as though forskolin either improves or does not have an effect on cell viability at lower doses of LPS, while it decreases cell viability at higher doses of LPS. Although cAMP is known to promote Schwann cell proliferation [9], results from an unpublished study have shown that different doses of forskolin at various time points have variable effects on Schwann cell viability. For example, in S16 Schwann cells, 0.5 μM of forskolin decreased viability after a 4-hour incubation time while it improved viability after a 12-hour incubation time, compared to the control. However, 3 μM of forskolin improved viability after a 12-hour incubation time while it decreased viability after a 24-hour incubation time (S2 Fig). Therefore, it is plausible that forskolin might decrease viability in certain treatment groups while improving viability in others. Future research should explore the dose- and time-dependent effects of forskolin on LPS-treated RT4-D6P2T cells to determine the optimal dose and time at which forskolin improves viability in a simulated inflammatory environment.

In addition to forskolin’s dose- and time-dependent effects, it is possible that it might also have variable effects on viability in different cell types and culture conditions. For instance, one study found that forskolin decreased viability in astrocytes subjected to oxidative stress, as indicated by a decrease in phosphorylated Akt (phospho-Akt) levels and an increase in caspase-3 levels [12]. In light of these findings, future studies could measure changes in phospho-Akt and caspase-3 expression to determine whether cAMP alters viability in RT4-D6P2T cells via phospho-Akt- and/or caspase-3-related pathways. An increased understanding of cAMP’s effects on RT4-D6P2T cells might help elucidate the mechanism by which cAMP regulates Schwann cell viability, or proliferation, during nerve injury and inflammation.

Additionally, it was observed that forskolin-mediated cAMP activation appears to alter the expression of proteins downstream of the NF-κB pathway in RT4-D6P2T cells treated with different doses of LPS. First, forskolin downregulated NF-κB expression in cells treated with or without 10 μg/mL of LPS, which was not unexpected because cAMP has been shown to inhibit the NF-κB pathway [13,14]. However, the same effect was not observed in cells treated with 0.1 and 1 μg/mL of LPS. Perhaps 2 μM of forskolin for a 3-hour incubation period was not sufficient to downregulate NF-κB in these cells. In future studies, a forskolin dose response should be performed to identify the optimal dose of forskolin for NF-κB activation.

In addition to NF-κB, forskolin also downregulated TNF-α expression in cells without LPS while it upregulated TNF-α expression in cells treated with LPS, compared to the control. The most striking increase in expression was observed in cells treated with 0.1 μg/mL of LPS. Considering TNF-α is produced downstream of the NF-κB pathway, it was expected that a decrease in NF-κB expression would be accompanied by a decrease in TNF-α, although this was not the case. However, it is not changes in NF-κB expression that alter TNF-α expression but, rather, changes in the nuclear translocation of NF-κB [5]. Once it makes its way from the cytoplasm and into the nucleus, NF-κB binds to genes responsible for the production of various cytokines, such as TNF-α [5,7,8]. It has been demonstrated that cAMP promotes the nuclear translocation of NF-κB via PKA and A kinase interacting protein 1 (AKIP1) in human embryonic kidney (HEK) cells [15]. In our study, forskolin-mediated cAMP activation might have also promoted the nuclear translocation of NF-κB, which would explain why forskolin upregulated TNF-α expression despite the downregulation of NF-κB expression in LPS-treated Schwann cells. Immunofluorescence studies could be conducted to determine whether forskolin-mediated cAMP activation alters the nuclear translocation of NF-κB in RT4-D6P2T cells treated with different doses of LPS. Likewise, the nuclear versus cytoplasmic expression of NF-κB should be quantified under different treatment conditions. This knowledge will provide insight into the effects of cAMP activation on the NF-κB signaling pathway in Schwann cells.

In regard to TNF-α, although we detected expression in LPS-treated RT4-D6P2T cells with or without cAMP activation, the form(s) of TNF-α that were being expressed are unknown. TNF-α exists in two different forms: transmembrane TNF-α and soluble TNF-α [16]. Transmembrane TNF-α is a 26 kDa protein located in the plasma membrane [16,17]. Once transmembrane TNF-α is cleaved by proteases, it becomes soluble TNF-α, a 15 kDa protein that gets secreted by the cell and mediates the immune response [16,17]. In this study, immunoblotting experiments using RT4-D6P2T cell lysates solely detected TNF-α expression approximately at the 26 kDa mark. It is plausible that either the RT4-D6P2T cells expressed transmembrane TNF-α but not soluble TNF-α, or soluble TNF-α expression was undetectable. However, because immunoblotting was performed using prepared RT4-D6P2T cell lysates, which contained processed proteins from both the cytoplasm and the plasma membrane, we cannot confirm that the TNF-α being expressed was only of the transmembrane form. Immunoblotting might have also detected cytoplasmic pro-TNF-α, transmembrane TNF-α that has yet to become anchored to the plasma membrane [17].

Future studies are necessary in order to identify the form(s) of TNF-α being expressed by RT4-D6P2T cells, their respective functions, and the mechanism by which they can exert their effects. Although transmembrane TNF-α and soluble TNF-α are able to bind to TNF receptors 1 and 2 (TNFR1 and TNFR2), transmembrane TNF-α appears to bind primarily to TNFR2, while soluble TNF-α appears to bind primarily to TNFR1 [18]. Transmembrane TNF-α is considered a bipolar molecule due to its ability to mediate forward and reverse signaling between the cell and its target cell [18]. In tumor cells, this membrane-bound form of TNF-α has been found to regulate cell survival depending on the direction of the signal. In forward signaling, transmembrane TNF-α acts as a ligand by binding to TNFR2, which in turn downregulates NF-κB activity and promotes apoptosis [18]. In reverse signaling, on the other hand, transmembrane TNF-α acts as a receptor to upregulate NF-κB activity and inhibit apoptosis [18]. In this study, since cAMP activation appears to regulate viability in LPS-treated RT4-D6P2T cells, we speculate whether cAMP’s effects were mediated by transmembrane TNF-α/TNFR2 activity. To answer this question, RT4-D6P2T cells can be treated with a TNFR2 inhibitor and the different doses of LPS, with or without forskolin, followed by a CellTiter-Glo viability assay to determine whether blocking TNFR2 activity affects cAMP’s ability to alter cell viability. This will further our knowledge of the potential interactions between the cAMP, NF-κB, and TNF-α signaling pathways, to regulate Schwann cell survival.

In addition to TNF-α expression, this study explored the effects of forskolin-mediated cAMP activation on TNF-α secretion in RT4-D6P2T cells. Although forskolin increased TNF-α expression at all LPS doses, it increased secretion in cells treated with lower doses of LPS (0.1 μg/mL) but decreased secretion in cells treated with higher doses of LPS (1 and 10 μg/mL). The ability of Schwann cells to regulate TNF-α secretion might be a potential mechanism by which Schwann cells mediate inflammation in the neuron microenvironment following nerve injury. It is possible that TNF-α has the ability to exert different effects at lower doses than at higher doses. A study conducted by Qin et al. investigated the dose-dependent effects of TNF-α on the expression of cytokines produced downstream of the NF-κB pathway in primary rat Schwann cells. It was found that when cells were treated with 10 ng/mL of TNF-α, this favored the expression of IL-10, an anti-inflammatory cytokine, whereas when cells were treated with 100 ng/mL of TNF-α, this favored the expression of IL-6, a proinflammatory cytokine [8]. These findings suggest that TNF-α might act to suppress inflammation at low concentrations while enhancing inflammation at high concentrations. Therefore, in this study, perhaps LPS-treated Schwann cells with cAMP activation secreted less TNF-α than cells without cAMP activation to upregulate IL-10 expression and, thus, counteract the effects of NF-κB-mediated inflammation.

Since cAMP activation appears to have an effect on TNF-α expression, we speculate whether cAMP activation also has an effect on the expression of IL-10 and IL-6, two cytokines that are also produced downstream of the NF-κB pathway. One study explored the effects of forskolin-mediated cAMP activation on IL-10 and IL-6 expression in LPS-treated microglial cells [19]. It was found that forskolin increased IL-10 expression while it decreased IL-6 expression compared to the control [19]. In light of these results, future studies could be performed to evaluate whether forskolin has similar effects on IL-10 and IL-6 expression in LPS-treated RT4-D6P2T cells.

Although it has been well-established that cAMP regulates cytokine expression downstream of the NF-κB pathway, the exact mechanism is unknown. One potential mechanism is through the differential expression of suppressors of cytokine signaling (SOCS) proteins 1 and 3, which have both been shown to suppress inflammation and, thus, appear to have neuroprotective effects. For instance, Kim et al. demonstrated that in LPS-treated macrophages overexpressing either the SOCS1 or SOCS3 gene, there was a significant reduction in TNF-α and IL-6 production [20]. However, in JEG-3 trophoblast cells overexpressing the SOCS3 gene, there was a significant increase in IL-10 production [21]. Furthermore, SOCS3 appears to mediate the inhibitory effects of IL-10 on TNF-α production in macrophages [22]. Therefore, although there is not much known regarding the role of SOCS1 and SOCS3 in Schwann cells, these two proteins might shed light on the complex interactions between cAMP and various cytokines produced downstream of the NF-κB pathway. In addition to IL-10 and IL-6 expression, future studies should measure SOCS1 and SOCS3 expression in LPS-treated RT4-D6P2T cells, with or without forskolin, to determine whether SOCS proteins mediate the effects of cAMP on cytokine expression. These findings might elucidate one of the potential mechanisms by which the cAMP pathway regulates NF-κB-mediated inflammation in Schwann cells during nerve injury and repair.

To explore the potential mechanisms by which cAMP regulates viability and the NF-κB signaling pathway in Schwann cells, we measured changes in the expression of two proteins downstream of the cAMP pathway: AKAP95 and cyclin D3. We observed striking differences in expression between forskolin-treated cells with 0.1 and 10 μg/mL of LPS. At 0.1 μg/mL of LPS, forskolin upregulated AKAP95 and cyclin D3 expression, while cell viability remained the same. This was unexpected because other studies have demonstrated the proliferative effects of AKAP95 and cyclin D3 in Schwann cells, although these were primary Schwann cells [9]. Perhaps at lower doses of LPS, NF-κB hinders the proliferative effects of AKAP95 and cyclin D3, which might be attributable to the apparent lack of change in cell viability. Conversely, at 10 μg/mL of LPS, forskolin downregulated AKAP95 and cyclin D3 expression, which correlated to a decrease in cell viability. Perhaps at higher doses of LPS for 3 hours, NF-κB hinders cAMP’s ability to upregulate AKAP95 and cyclin D3, attributing to a decrease in cell viability. Future studies are required to determine exactly how different doses of LPS alter the expression of AKAP95 and cyclin D3 in forskolin-treated RT4-D6P2T cells.

Furthermore, considering the potential role of AKAP95 in cAMP-mediated TNF-α suppression, we performed a Pearson’s correlation to determine whether an increase in AKAP95 expression was associated with a decrease in TNF-α expression. We found that there was no significant association between AKAP95 and TNF-α expression. Likewise, there was no significant association between cyclin D3 and TNF-α expression.

**Fig 7:**
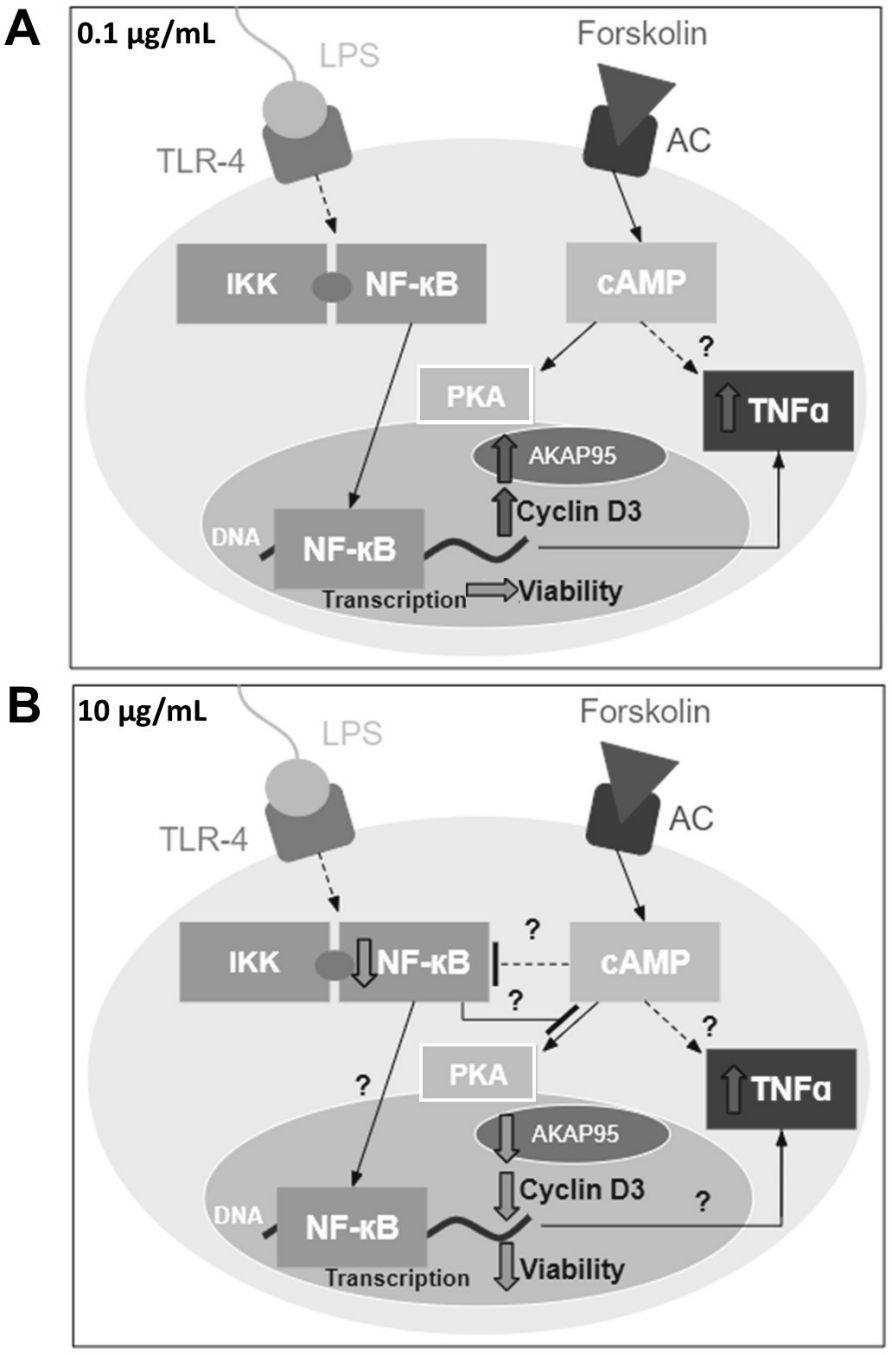
Summary of the effects of forskolin-mediated cAMP activation on protein expression and viability in Schwann cells. Cartoon illustration depicting the effects of forskolin-mediated cAMP activation on NF-κB, TNF-α, AKAP95, and cyclin D3 expression, and viability in Schwann cells treated with (A) 0.1 and (B) 10 μg/mL of LPS for 3 hours.

In summary, as depicted in Fig 7, forskolin-mediated cAMP activation appears to have variable effects on protein expression and viability in Schwann cells treated with different doses of LPS for 3 hours. At 0.1 μg/mL of LPS, cAMP activation did not alter NF-κB expression, but it upregulated TNF-α. At 10 μg/mL of LPS, cAMP activation continued to upregulate TNF-α expression despite the downregulation of NF-κB. These results provide evidence that the cAMP pathway might upregulate TNF-α expression through other pathways. Furthermore, at 0.1 μg/mL of LPS, cAMP activation upregulated the expression of AKAP95 and cyclin D3, while it did not affect cell viability. However, at 10 μg/mL of LPS, cAMP activation downregulated the expression of AKAP95 and cyclin D3 and impaired cell viability. We speculate that at low doses of LPS, NF-κB might hinder the proliferative effects of AKAP95 and cyclin D3. On the other hand, at high doses of LPS, NF-κB might hinder cAMP’s ability to upregulate the expression of AKAP95 and cyclin D3, resulting in decreased cell viability.

Although the cAMP and NF-κB pathways, and their individual roles in inflammation, in Schwann cells have been previously studied, this study suggests that the possible interactions between these two pathways cannot be overlooked. We believe that identifying the signaling effectors that coordinate these two pathways will not only increase our understanding of Schwann cells’ function in nerve injury and repair, but it will also shed light on a potential therapeutic target for the treatment of nerve injury and inflammation.

## Materials and methods

### Antibodies and reagents

For the viability assay, the CellTiter-Glo 2.0 Assay kit was purchased from Promega (Cat #G9243, Madison, WI). For immunoblotting, the following antibodies were purchased: anti-rat NF-κB p65 (dilution– 1:750, Thermo Fisher, Cat #436700, Waltham, MA), anti-TNF-α (dilution– 1:1000, Thermo Fisher, Cat #BMS175, Waltham, MA), anti-AKAP95 (dilution– 1:2500, gifted by Vince Coghlan, OHSU, Portland, OR), anti-cyclin D3 (dilution– 1:1000, BD Biosciences, Cat #610279, Franklin Lakes, NJ), and anti-actin (dilution– 1:1000, Sigma-Aldrich, Cat #A2066, St. Louis, MO). For TNF ELISA, the Rat TNF-alpha ELISA kit was purchased from RayBiotech (Cat #ELR-TNFa-1, Norcross, GA).

### Cell culture

The immortalized rat RT4-D6P2T Schwann cell line (ATCC, Cat #CRL-2768, Manassas, VA) and S16 Schwann cell line (ATCC, Cat #CRL-2941, Manassas, VA) were aseptically cultured and subcultured (at 80% confluency) in Dulbecco’s Modified Eagle Medium (DMEM) (ATCC, Cat #30–2002, Manassas, VA) supplemented with 10% fetal bovine serum (FBS) (Thermo Fisher, Cat #16000044, Waltham, MA) and 1% penicillin/streptomycin (Pen-strep) (GIBCO, Cat #15140–015, Gaithersburg, MD)/amphotericin B (R&D Systems, Cat #B23192, Minneapolis, MN) at 37°C and 5% CO_2_ in poly-L-lysine-coated dishes. For all cells treated with LPS, lipopolysaccharide (LPS) from *Escherichia coli* O111:B4 (Sigma, Cat #L2630, St. Louis, MO) was reconstituted in 1 mL of autoclaved deionized water for a concentration of 1 mg/mL and further diluted depending on the required dose for each experiment.

### Cell viability assay

Upon reaching 80% confluency, the RT4-D6P2T and S16 cells were seeded separately into DMEM in poly-L-lysine-coated 96-well plates at a density of 20,000 cells per well. After a 24-hour incubation with DMEM, all the DMEM was aspirated from the wells and replaced with colorless N_2_ media (DMEM/F12, no phenol red [Thermo Fisher, Cat #21041025, Waltham, MA] supplemented with 5 μg/mL insulin [Sigma, Cat #91077C, St. Louis, MO] and 100 μg/mL apo-transferrin [Sigma, Cat #T1147, St. Louis, MO]). After a 24-hour incubation, the N_2_ was aspirated from the wells. Based on preliminary studies, the cells received one of the following treatments: 0.1, 1, or 10 μg/mL of LPS, in N_2_ media (control) or N_2_ media supplemented with 2 μM of forskolin (Sigma, Cat #F-6886, St. Louis, MO), for 1, 3, 12, or 24 hours. After the cultures were incubated in the different treatments for the appropriate amount of time, the CellTiter-Glo 2.0 Assay kit (Promega) was used to perform the viability assay. The plate and cell contents were equilibrated for approximately 30 minutes. Then a volume of CellTiter-Glo 2.0 Reagent equivalent to the volume of cell culture medium was added to each well. The contents were mixed for 2 minutes to induce cell lysis. Once the contents were mixed and equilibrated, luminescence was read in the SpectraMax M4 Multi-Mode Microplate Reader (Molecular Devices, San Jose, CA), and data were collected using SoftMax V3.0 software. Luminescence was measured as an indicator of cell viability, with a higher luminescence indicating more viable cells, and a lower luminescence indicating less viable cells. The data are presented as a mean percent control to show the effects of the forskolin treatment on the viability of cells treated with different doses of LPS.

doi: https://doi.org/10.17504/protocols.io.yxmvm3815l3p/v1

### Immunoblotting

Upon reaching 80% confluency, the RT4-D6P2T cells were seeded into DMEM in poly-L-lysine-coated 6-well plates at a density of 300,000 cells per well. After a 24-hour incubation with DMEM, all the DMEM was aspirated from the wells and replaced with colorless N_2_ media. After a 24-hour incubation, the N_2_ was aspirated from the wells. Based on preliminary studies, the cells received one of the following treatments: 0.1, 1, or 10 μg/mL of LPS, in N_2_ media (control) or N_2_ media supplemented with 2 μM of forskolin, for 3 hours. After 3 hours, media samples were collected, and cell lysates were prepared using IP lysis buffer (Thermo Fisher, Cat #87787, Waltham, MA) supplemented with 10% 100X protease inhibitor cocktail (Sigma, Cat #P8849, St. Louis, MO). SDS-PAGE gel electrophoresis and Western blotting were performed using the prepared cell lysates. For all proteins, different replicates and treatment combinations were run on different gels. Individual membrane strips were cut and probed for Western blotting. The membranes were incubated in primary antibodies against NF-κB, TNF-α, AKAP95, or cyclin D3, diluted in 5% milk for 1 hour at room temperature or overnight at 4°C. β-actin was used as a loading control. Detected expression levels of actin were used to normalize all Western blots. The membranes were then incubated in horseradish peroxidase-conjugated secondary antibodies diluted in 5% milk for 1 hour at room temperature. Protein expression was visualized using SuperSignal West Pico PLUS Chemiluminescent Substrate (Thermo Fisher, Cat #34580, Waltham, MA) and quantified via densitometry analysis using Image Lab 6.1 software (Bio-Rad Laboratories, Hercules, CA). The data are presented as mean fold change (over the N_2_ control [no LPS]) to show the effects of the forskolin treatment on NF-κB, TNF-α, AKAP95, and cyclin D3 expression in cells treated with different doses of LPS.

doi: https://doi.org/10.17504/protocols.io.yxmvm3dwnl3p/v1

### TNF ELISA

Media samples were collected from the 6-well plates (as described above), and the Rat TNF-alpha ELISA kit (RayBiotech, Cat #ELR-TNFa-1, Norcross, GA) was used to quantify the amount of TNF-α secreted by the RT4-D6P2T cells in response to the following treatments: 0.1, 1, or 10 μg/mL of LPS, in N_2_ media (control) or N_2_ media supplemented with 2 μM of forskolin, for 3 hours. The samples were diluted two-fold, and the ELISA was conducted following the manufacturer’s protocol. Immediately after the final incubation, the 96-well plate was read at 450 nm in the SpectraMax ABS Microplate Reader (Molecular Devices, San Jose, CA). TNF-α levels were calculated based on the standard curve, and the data are presented as mean fold change (over the N_2_ control [no LPS]) to show the effects of the forskolin treatment on TNF-α secretion by cells treated with different doses of LPS.

doi: https://doi.org/10.17504/protocols.io.3byl4qp88vo5/v1.

### Statistical analysis

The results from the viability assay, immunoblotting, and TNF ELISA were obtained from three independent experiments. Statistical analysis was conducted using R Statistical Software (v4.2.2; R Core Team 2022), and figures were produced using the ggplot2 R package (v3.4.2; Wickham 2016). For the cell viability assay, one-way analysis of variance (ANOVA) was used to compare mean percent control between the different treatment groups. Significant values (*p* < 0.05) were further examined using Tukey’s post-hoc and least significant difference (LSD) tests. Pearson’s correlation was used to determine if there is a linear relationship between time and cell viability and between LPS dose and cell viability. For immunoblotting, one-way ANOVA was used to compare mean fold change between the different treatment groups. Significant values (*p* < 0.05) were further examined using Tukey’s and LSD tests. For TNF ELISA, one-way ANOVA was used to compare mean fold change between the different treatment groups. Significant values (*p* < 0.05) were further examined using Tukey’s and LSD tests. Pearson’s correlation was used to determine if there is a linear relationship between NF-κB expression and TNF-α expression, AKAP95 expression and TNF-α expression, cyclin D3 expression and TNF-α expression, and TNF-α expression and TNF-α secretion.

## Supporting information

S1 Fig The effects of forskolin on the viability of LPS-treated S16 cells. Using the CellTiter-Glo 2.0 Assay (Promega), the immortalized rat S16 cell line (ATCC #CRL-2941) was treated with 0.1, 1, or 10 μg/mL of LPS in N_2_ media, with or without 2 μM of forskolin, for (A) 1, (B) 3, (C) 12, and (D) 24 hours. Relative luminescence units were read as an indicator of viability and are displayed as a mean percent control ± SEM. The dotted line indicates a percent control of 100%, with a percent control above 100% representing increased relative luminescence units (more viable cells) and a percent control below 100% representing decreased relative luminescence units (less viable cells) compared to the N_2_ control. Results from all experiments were examined using one-way ANOVA and tested with Tukey’s and LSD post-hoc analysis. Mean percent controls with the same number of asterisks are significantly different from each other (*p < 0.05, **p < 0.01, ***p < 0.001) (n = 3).
(TIF)

S2 Fig Unpublished forskolin dose response study (Williams 2018). Using the CyQUANT^TM^ MTT Cell Viability Assay Kit (Thermo Fisher), the immortalized rat S16 cell line (ATCC #CRL-2941) was treated with 0.5, 1, 2, or 3 μM of forskolin for (A) 4, (B) 6, (C) 12, and (D) 24 hours. Optical density was read at 570 nm as an indicator of viability and is displayed as a mean percent control ± SEM. The dotted line indicates a percent control of 100%, with a percent control above 100% representing increased optical density (more viable cells) and a percent control below 100% representing decreased optical density (less viable cells) compared to the N_2_ control. Results from all experiments were examined using one-way ANOVA and tested with Tukey’s and LSD post-hoc analysis (n = 4).
(TIF)

S1 Table RT4-D6P2T cell viability *p*-values. Using R Statistical Software (v4.2.2; R Core Team 2022), the degree of difference between different treatments was determined by performing a series of one-way ANOVA and tested with Tukey’s and LSD post-hoc analysis (*p < 0.05).
(TIF)

S2 Table NF-κB, TNF-α, AKAP95, and Cyclin D3 expression *p*-values. Using R Statistical Software (v4.2.2; R Core Team 2022), the degree of difference between different treatments was determined by performing a series of one-way ANOVA and tested with Tukey’s and LSD post-hoc analysis (*p < 0.05).
(TIF)

S1 Data Raw data file for RT4-D6P2T cell viability assay. This Supporting Information file contains all the raw data and figures from the RT4-D6P2T cell viability assay experiments. The data are presented as luminescence values (left) and percent control (right). The data are also grouped by incubation time (1 hour, 3 hours, 12 hours, and 24 hours), media type (N2 [control media] and FSK [forskolin-supplemented media]), and LPS dose (0 LPS, 0.1 LPS, 1 LPS, and 10 LPS).
(XLSX)

S2 Data Raw data file for S16 cell viability assay. This Supporting Information file contains all the raw data and figures from the S16 cell viability assay experiments. The data are presented as luminescence values (left) and percent control (right). The data are also grouped by incubation time (1 hour, 3 hours, 12 hours, and 24 hours), media type (N2 [control media] and FSK [forskolin-supplemented media]), and LPS dose (0 LPS, 0.1 LPS, 1 LPS, and 10 LPS).
(XLSX)

S3 Data Original uncropped and unadjusted blot images. This Supporting Information file contains all the original uncropped and unadjusted blot images from the immunoblotting experiments. An “X” above the lane indicates that the blots in that particular lane were not included in the results.
(PDF)

S4 Data Raw data file for TNF ELISA. This Supporting Information file contains all the raw data from the TNF ELISA experiments using RT4-D6P2T cells. The data are grouped by LPS dose and whether the cells received the forskolin treatment. The data are presented as TNF-α concentration (pg/mL) and fold change.
(XLSX)

## References

[1] Martini R, Fischer S, López-Vales R, David S. Interactions between Schwann cells and macrophages in injury and inherited demyelinating disease. Glia. 2008;56(14): 1566–1577. doi: 10.1002/glia.20766 18803324

[2] Bacallao K, Monje PV. Opposing roles of PKA and EPAC in the cAMP-dependent regulation of Schwann cell proliferation and differentiation. PLoS ONE. 2013;8(12): e82354. doi: 10.1371/journal.pone.0082354 24349260 PMC3859537

[3] Knott EP, Assi M, Pearse DD. Cyclic AMP signaling: A molecular determinant of peripheral nerve regeneration. Biomed Res Int. 2014: 651625. doi: 10.1155/2014/651625 25177696 PMC4142170

[4] Lu Y, Yeh W, Ohashi PS. LPS/TLR4 signal transduction pathway. Cytokine. 2008;42(2): 145–151. doi: 10.1016/j.cyto.2008.01.006 18304834

[5] Qin Y, Hua M, Duan Y, Gao Y, Shao X, Wang H, et al. TNF-α expression in Schwann cells is induced by LPS and NF-κB-dependent pathways. Neurochem Res. 2012;37(4): 722–731. doi: 10.1007/s11064-011-0664-2 22219126

[6] Wagner R, Myers RR. Schwann cells produce tumor necrosis factor alpha: Expression in injured and non-injured nerves. Neuroscience. 1996;73(3): 625–629. doi: 10.1016/0306-4522(96)00127-3 8809782

[7] Cheng C, Qin Y, Shao X, Wang H, Gao Y, Cheng M, et al. Induction of TNF-alpha by LPS in Schwann cell is regulated by MAPK activation signals. Cell Mol Neurobiol. 2007;27(7): 909–921. doi: 10.1007/s10571-007-9215-4 17902045 PMC11517400

[8] Qin Y, Cheng C, Wang H, Shao X, Gao Y, Shen A. TNF-alpha as an autocrine mediator and its role in the activation of Schwann cells. Neurochem Res. 2008;33(6): 1077–1084. doi: 10.1007/s11064-007-9552-1 18205044

[9] Asirvatham AL, Schworer CM, Stahl R, Heitzman D, Carey DJ. Role of A-kinase anchoring proteins in cyclic-AMP-mediated Schwann cell proliferation. Cell Signal. 2021;83: 109977. doi: 10.1016/j.cellsig.2021.109977 33716104

[10] Wall EA, Zavzavadjian JR, Chang MS, Randhawa B, Zhu X, Hsueh RC, et al. Suppression of LPS-induced TNF-alpha production in macrophages by cAMP is mediated by PKA-AKAP95-p105. Sci Signal. 2009;2(75): ra28. doi: 10.1126/scisignal.2000202 19531803 PMC2770900

[11] Harper CV, Woodcock DJ, Lam C, Garcia-Albornoz M, Adamson A, Ashall L, et al. Temperature regulates NF-κB dynamics and function through timing of A20 transcription. Proc Natl Acad Sci. 2018;115(22): E5243–E5249. doi: 10.1073/pnas.1803609115 29760065 PMC5984538

[12] Shim MS, Kim K, Bu JH, Nam HS, Jeong SW, Park TL, et al. Elevated intracellular cAMP exacerbates vulnerability to oxidative stress in optic nerve head astrocytes. Cell Death Dis. 2018;9(3): 285. doi: 10.1038/s41419-017-0171-8 29459737 PMC5833440

[13] Wen AY, Sakamoto KM, Miller LS. The role of the transcription factor CREB in immune function. J Immunol. 2010;185(11): 6413–6419. doi: 10.4049/jimmunol.1001829 21084670 PMC5519339

[14] Minguet S, Huber M, Rosenkranz L, Schamel WWA, Reth M, Brummer T. Adenosine and cAMP are potent inhibitors of the NF-kappaB pathway downstream of immunoreceptors. Eur J Immunol. 2005;35(1): 31–41. doi: 10.1002/eji.200425524 15580656

[15] King CC, Sastri M, Chang P, Pennypacker J, Taylor SS. The rate of NF-κB nuclear translocation is regulated by PKA and A kinase interacting protein 1. PLoS ONE. 2011;6(4): e18713. doi: 10.1371/journal.pone.0018713 21556136 PMC3083391

[16] Horiuchi T, Mitoma H, Harashima S, Tsukamoto H, Shimoda T. Transmembrane TNF-α: structure, function and interaction with anti-TNF agents. Rheumatology (Oxford). 2010;49(7): 1215–1228. doi: 10.1093/rheumatology/keq031 20194223 PMC2886310

[17] Deora A, Hegde S, Lee J, Choi C, Chang Q, Lee C, et al. Transmembrane TNF-dependent uptake of anti-TNF antibodies. mAbs. 2017;9(4): 680–695. doi: 10.1080/19420862.2017.1304869 28323513 PMC5419086

[18] Qu Y, Zhao G, Li H. Forward and reverse signaling mediated by transmembrane tumor necrosis factor-alpha and TNF receptor 2: Potential roles in an immunosuppressive tumor microenvironment. Front Immunol. 2017;8: 1675. doi: 10.3389/fimmu.2017.01675 29234328 PMC5712345

[19] Woo M, Jung S, Hyun J, Kim H. Differential regulation of inducible nitric oxide synthase and cytokine gene expression by forskolin and dibutyryl-cAMP in lipopolysaccharide-stimulated murine BV2 microglial cells. Neurosci Lett. 2004;356(3): 187–190. doi: 10.1016/j.neulet.2003.11.056 15036626

[20] Kim G, Jeong H, Yoon H, Yoo H, Lee JY, Park SH, et al. Anti-inflammatory mechanisms of suppressors of cytokine signaling target ROS via NRF-2/thioredoxin induction and inflammasome activation in macrophages. BMB Rep. 2020;53(12): 640–645. doi: 10.5483/BMBRep.2020.53.12.161 33172542 PMC7781909

[21] Dong Q, Fan R, Zhao S, Wang Y. Over-expression of SOCS-3 gene promotes IL-10 production by JEG-3 trophoblast cells. Placenta. 2009;30(1): 11–14. doi: 10.1016/j.placenta.2008.10.008 19036437 PMC3066079

[22] Qasimi P, Ming-Lum A, Ghanipour A, Ong CJ, Cox ME, Ihle J, et al. Divergent mechanisms utilized by SOCS3 to mediate interleukin-10 inhibition of tumor necrosis factor alpha and nitric oxide production by macrophages. J Biol Chem. 2006;281(10):6316–24. doi: 10.1074/jbc.M508608200 16352613

