# Supplementary figures and images for: Forskolin-mediated cAMP activation upregulates TNF-α expression despite NF-κB downregulation in LPS-treated Schwann cells
Source: PLoS One. 2024 Apr 16;19(4):e0302223. doi: 10.1371/journal.pone.0302223 (PMC11020835; doi:10.1371/journal.pone.0302223)

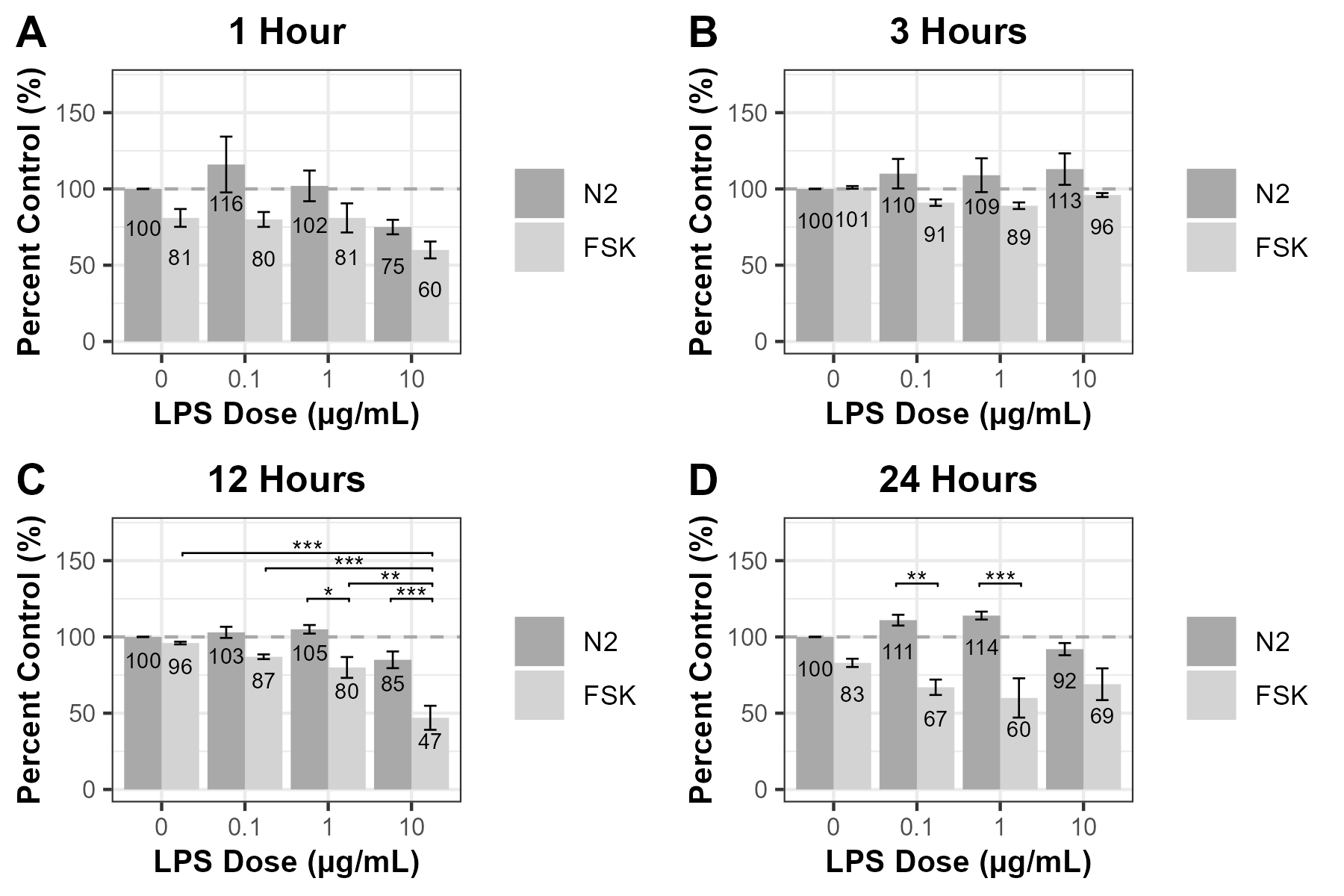

Supplement: S1 Fig — Using the CellTiter-Glo 2.0 Assay (Promega), the immortalized rat S16 cell line (ATCC #CRL-2941) was treated with 0.1, 1, or 10 μg/mL of LPS in N2 media, with or without 2 μM of forskolin, for (A) 1, (B) 3, (C) 12, and (D) 24 hours. Relative luminescence units were read as an indicator of viability and are displayed as a mean percent control ± SEM. The dotted line indicates a percent control of 100%, with a percent control above 100% representing increased relative luminescence units (more viable cells) and a percent control below 100% representing decreased relative luminescence units (less viable cells) compared to the N2 control. Results from all experiments were examined using one-way ANOVA and tested with Tukey’s and LSD post-hoc analysis. Mean percent controls with the same number of asterisks are significantly different from each other (*p < 0.05, **p < 0.01, ***p < 0.001) (n = 3). (TIF) [file pone.0302223.s001.tif]

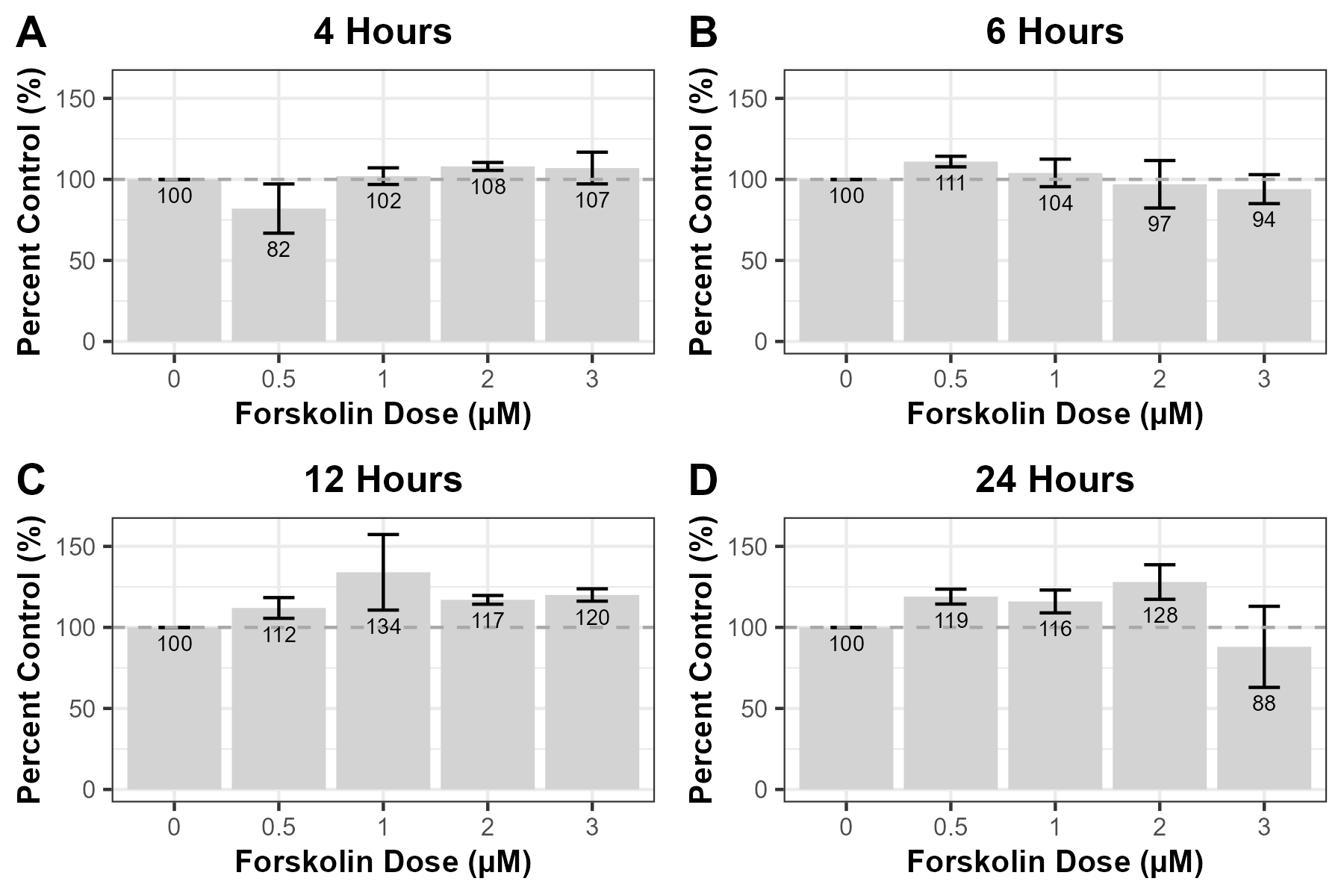

Supplement: S2 Fig — Using the CyQUANTTM MTT Cell Viability Assay Kit (Thermo Fisher), the immortalized rat S16 cell line (ATCC #CRL-2941) was treated with 0.5, 1, 2, or 3 μM of forskolin for (A) 4, (B) 6, (C) 12, and (D) 24 hours. Optical density was read at 570 nm as an indicator of viability and is displayed as a mean percent control ± SEM. The dotted line indicates a percent control of 100%, with a percent control above 100% representing increased optical density (more viable cells) and a percent control below 100% representing decreased optical density (less viable cells) compared to the N2 control. Results from all experiments were examined using one-way ANOVA and tested with Tukey’s and LSD post-hoc analysis (n = 4). (TIF) [file pone.0302223.s002.tif]

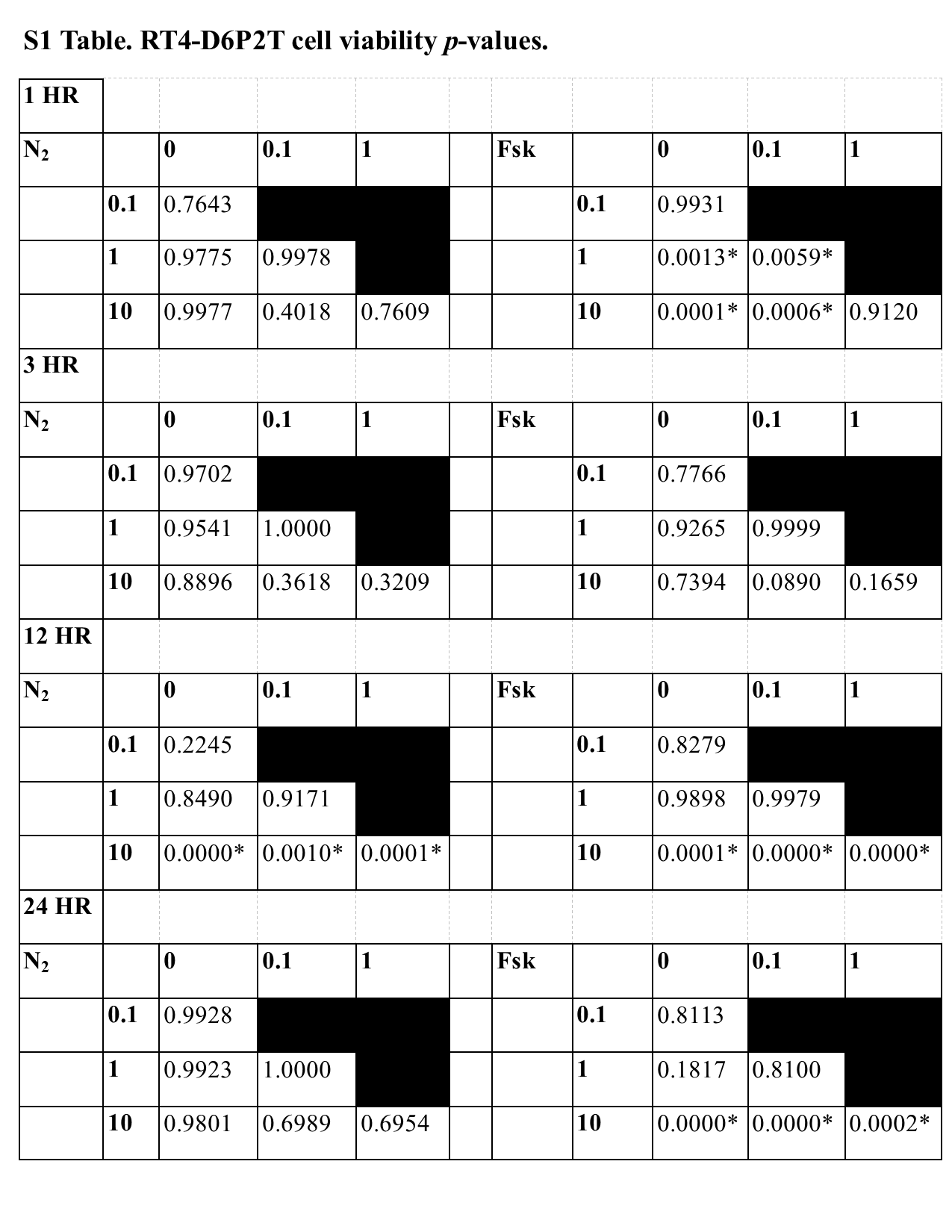

Supplement: S1 Table — Using R Statistical Software (v4.2.2; R Core Team 2022), the degree of difference between different treatments was determined by performing a series of one-way ANOVA and tested with Tukey’s and LSD post-hoc analysis (*p < 0.05). (TIF) [file pone.0302223.s003.tif]

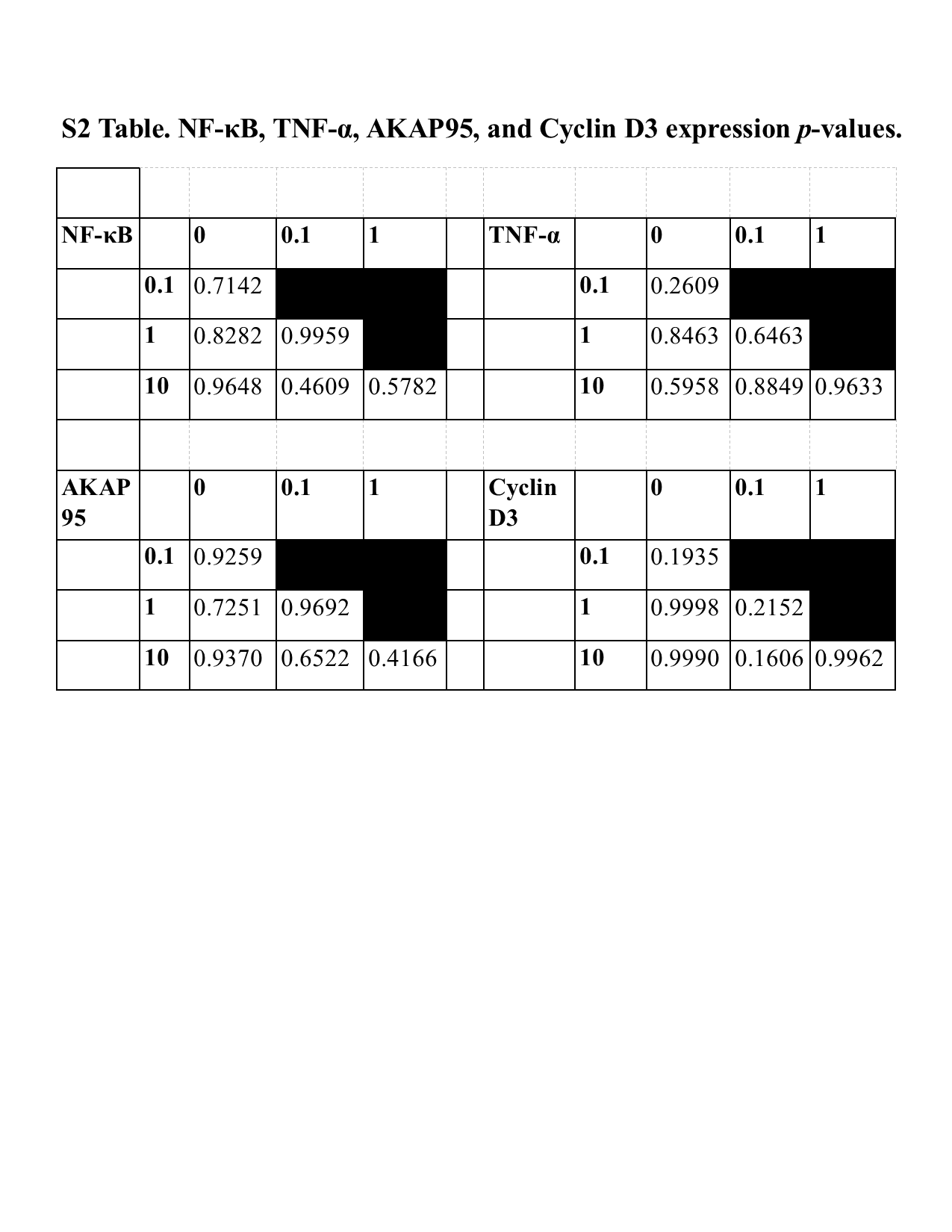

Supplement: S2 Table — Using R Statistical Software (v4.2.2; R Core Team 2022), the degree of difference between different treatments was determined by performing a series of one-way ANOVA and tested with Tukey’s and LSD post-hoc analysis (*p < 0.05). (TIF) [file pone.0302223.s004.tif]
