# Supplementary material for: Forskolin-mediated cAMP activation upregulates TNF-α expression despite NF-κB downregulation in LPS-treated Schwann cells
Source: PLoS One. 2024 Apr 16;19(4):e0302223. doi: 10.1371/journal.pone.0302223 (PMC11020835; doi:10.1371/journal.pone.0302223)
Supplement: S3 Data — This Supporting Information file contains all the original uncropped and unadjusted blot images from the immunoblotting experiments. An “X” above the lane indicates that the blots in that particular lane were not included in the results. (PDF) [file pone.0302223.s007.pdf]

0 µg/mL LPS

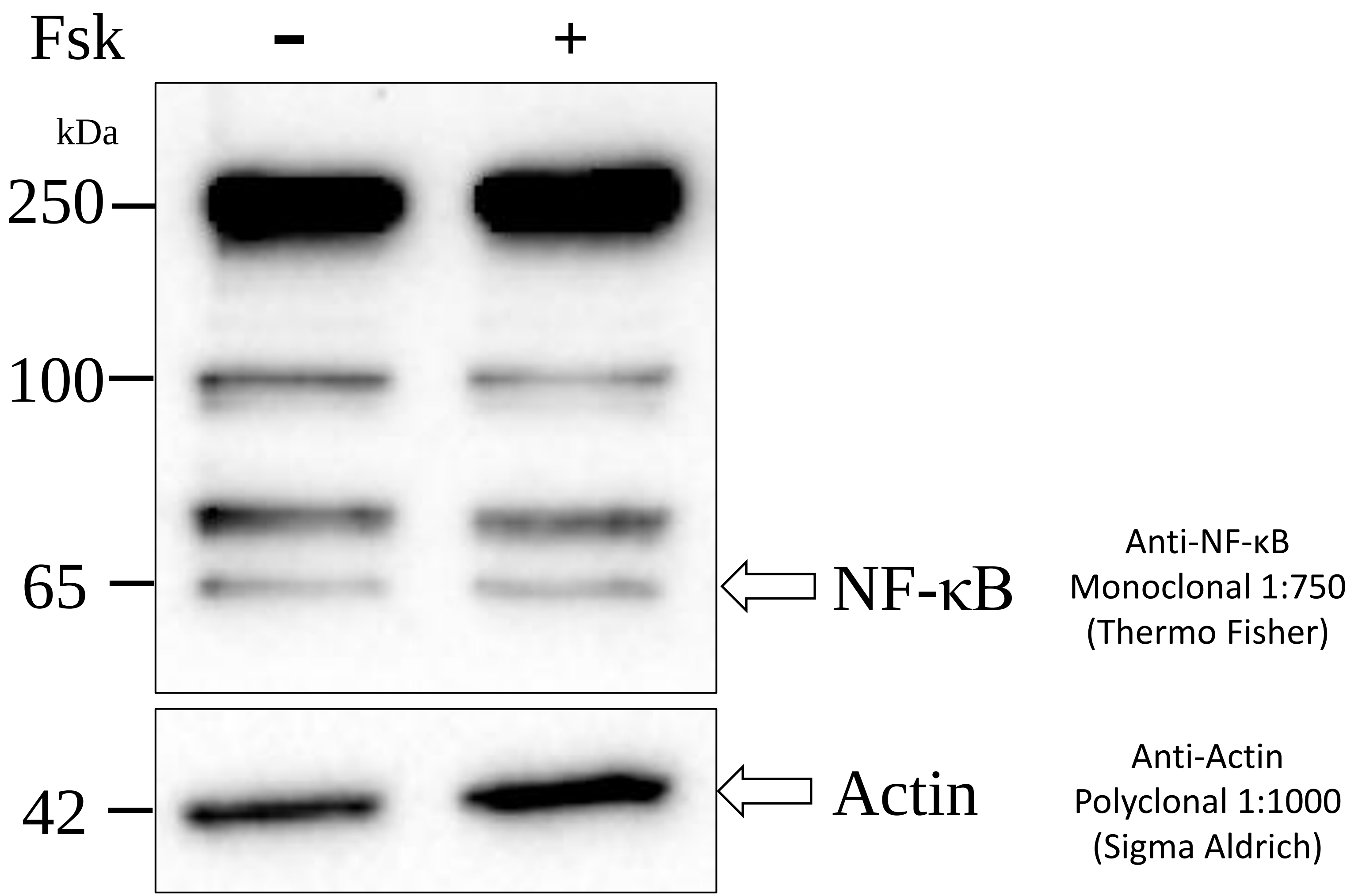

- **Date of sample preparation:** 9/12/22
- **Date of gel:** 9/13/22
- **Date of WB:** 9/14/22
- **Sample:** RT4-D6P2T cell lysates
- **Washes:** 3x 3-min washes in Blotto
- **Last wash:** 1x Blotto, 2x TTBS
- **Exposure time:** 50 sec (NF-κB); 10 sec (Actin)
- **Corresponding figure:** Figure 2A

0.1 μg/mL LPS

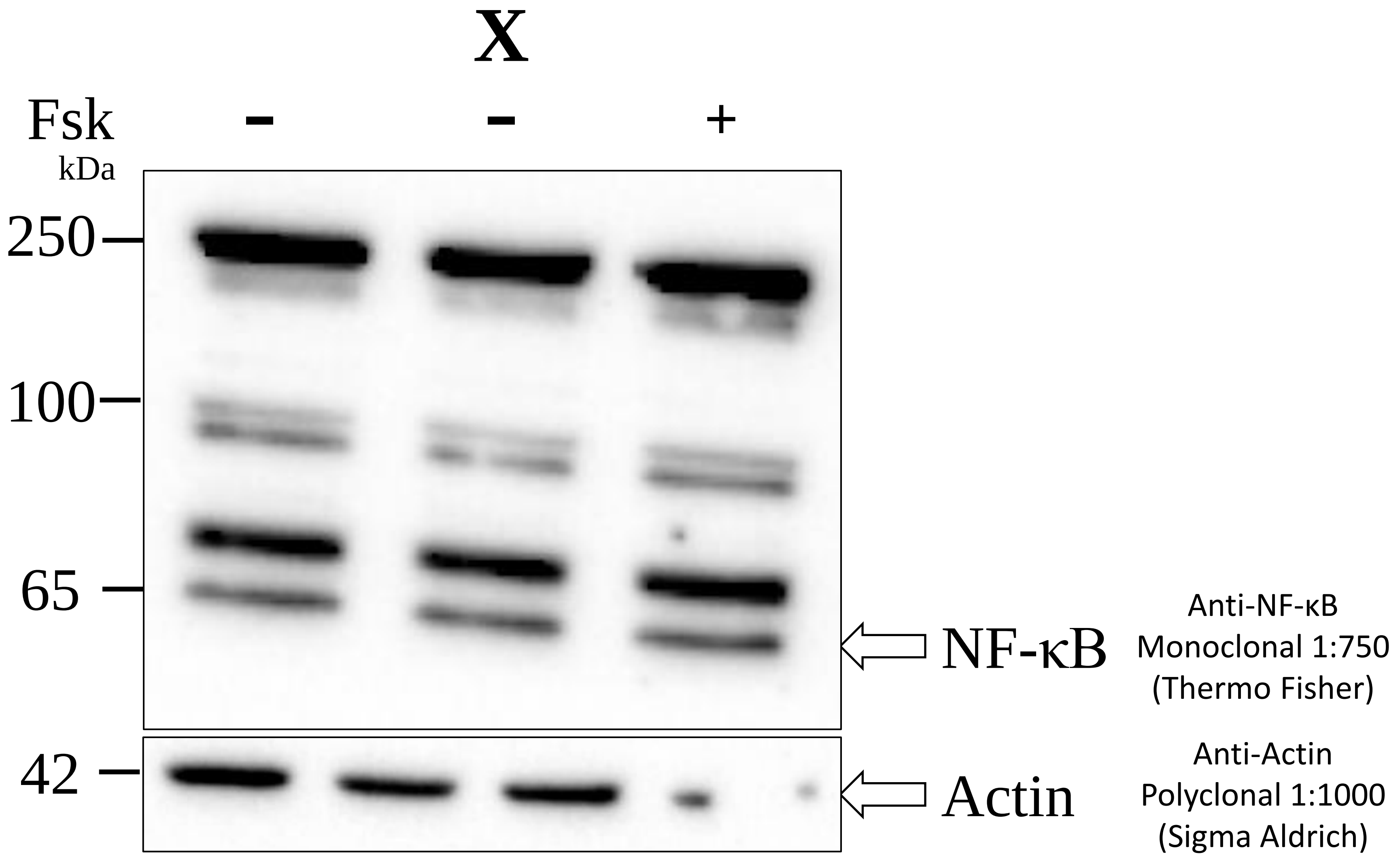

- **Date of sample preparation:** 9/18/22
- **Date of gel:** 9/20/22
- **Date of WB:** 9/21/22
- **Sample:** RT4-D6P2T cell lysates
- **Washes:** 3x 3-min washes in Blotto
- **Last wash:** 1x Blotto, 2x TTBS
- **Exposure time:** 25 sec (NF-κB); 7 sec (Actin)
- **Corresponding figure:** Figure 2A

1 μg/mL LPS

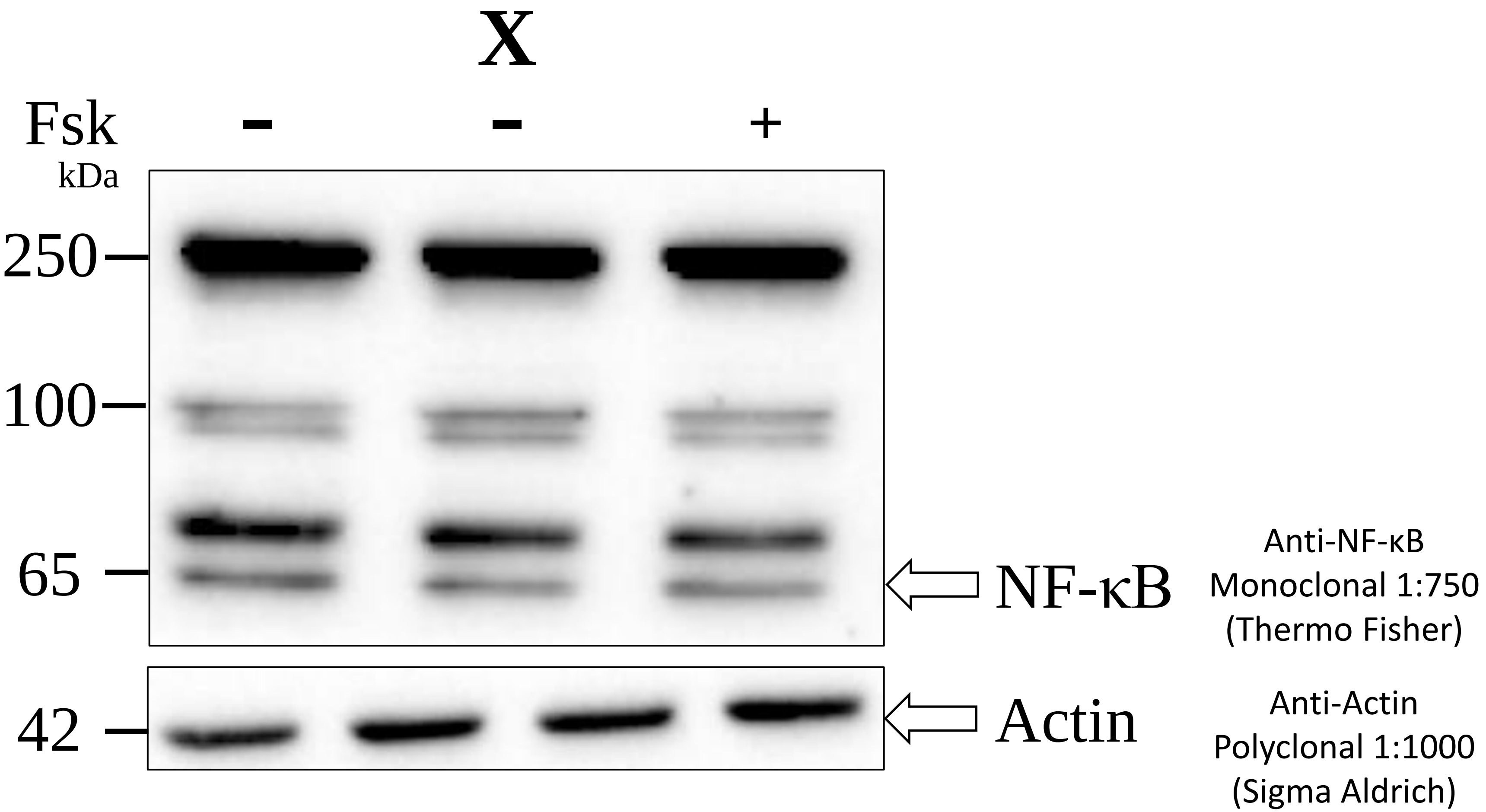

- **Date of sample preparation:** 9/18/22
- **Date of gel:** 9/20/22
- **Date of WB:** 9/21/22
- **Sample:** RT4-D6P2T cell lysates
- **Washes:** 3x 3-min washes in Blotto
- **Last wash:** 1x Blotto, 2x TTBS
- **Exposure time:** 25 sec (NF-κB); 3 sec (Actin)
- **Corresponding figure:** Figure 2A

10 μg/mL LPS

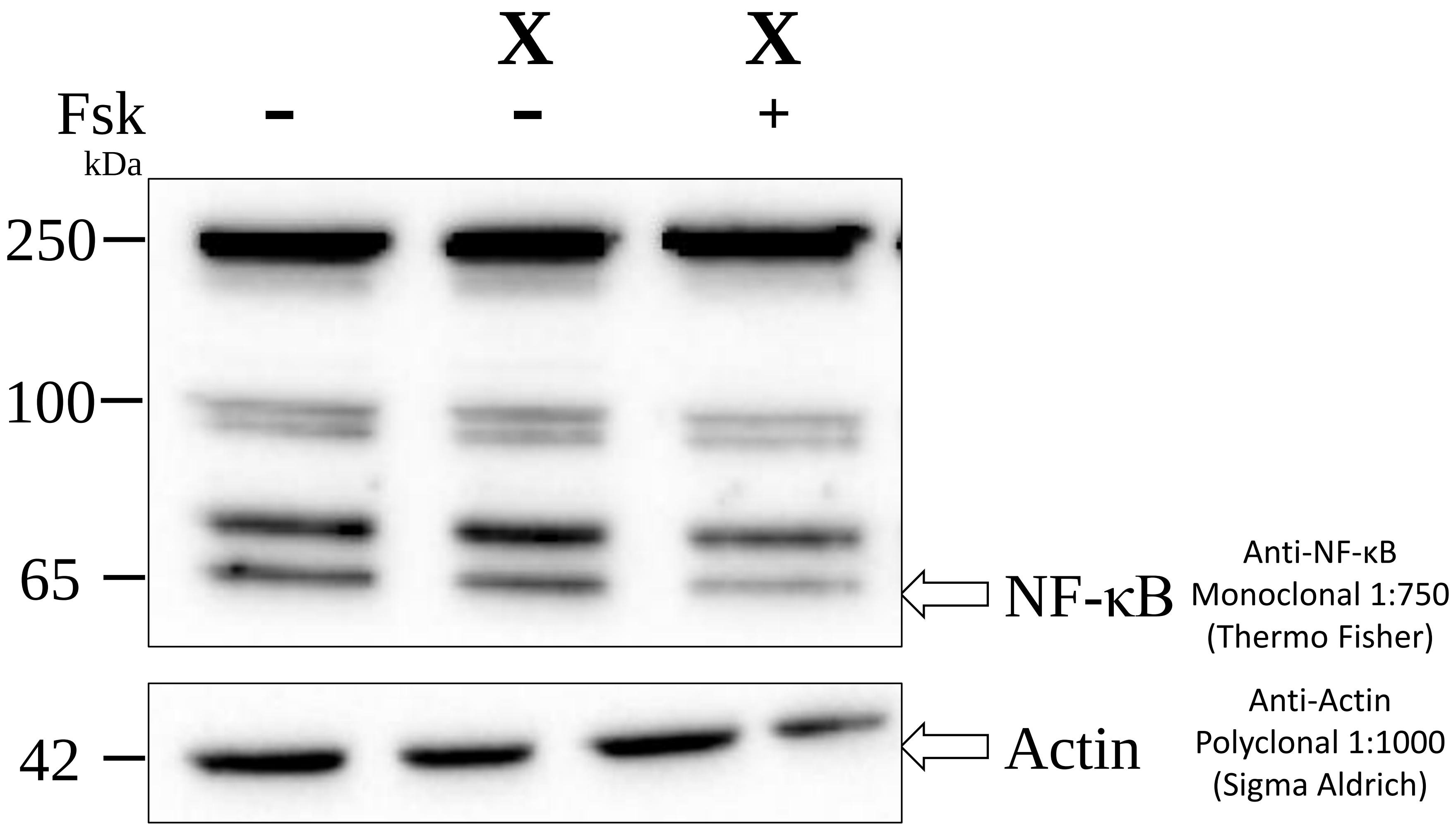

- **Date of sample preparation:** 9/18/22
- **Date of gel:** 9/20/22
- **Date of WB:** 9/21/22
- **Sample:** RT4-D6P2T cell lysates
- **Washes:** 3x 3-min washes in Blotto
- **Last wash:** 1x Blotto, 2x TTBS
- **Exposure time:** 20 sec (NF-κB); 4 sec (Actin)
- **Corresponding figure:** Figure 2A

10 μg/mL LPS

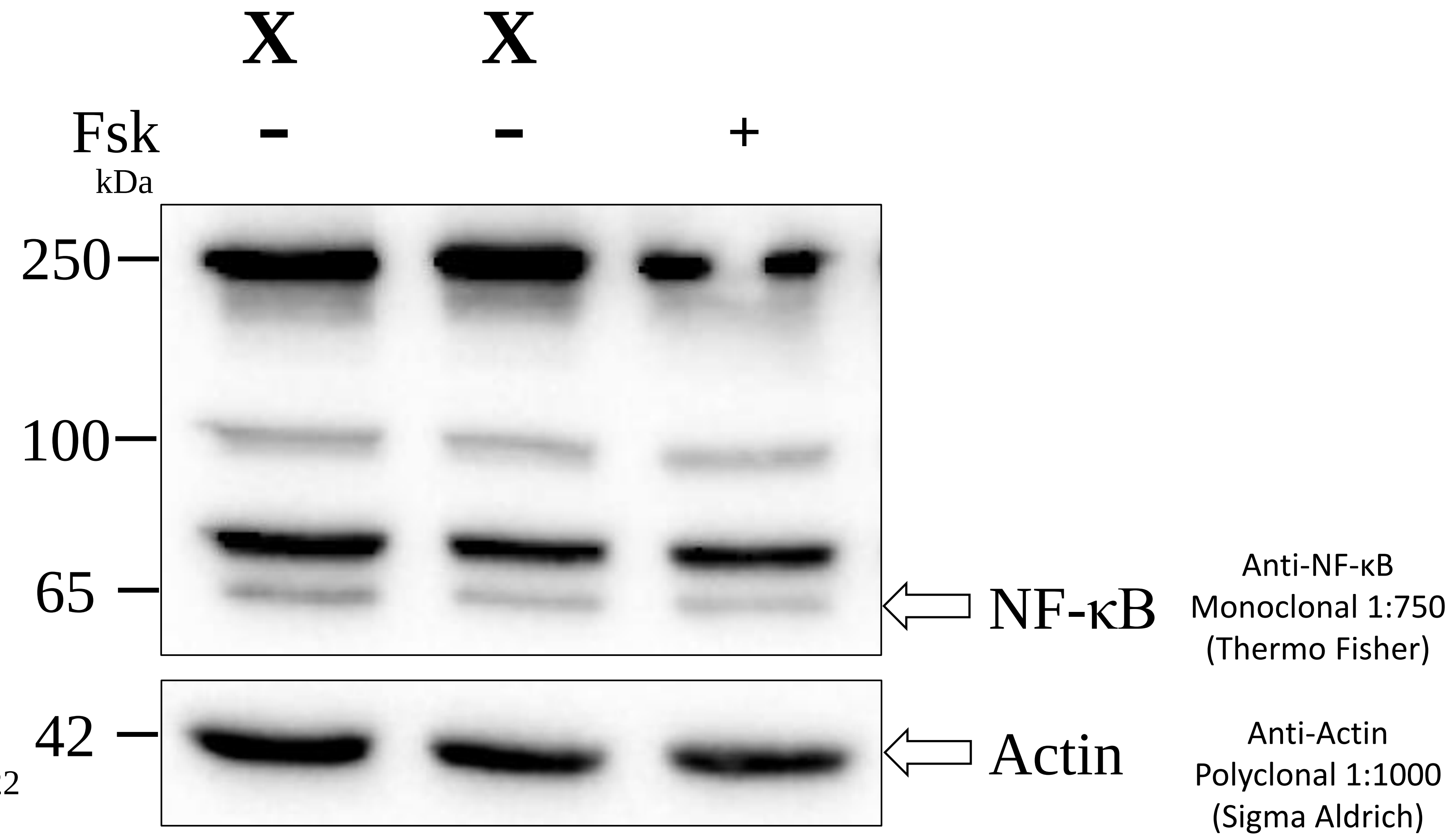

- **Date of sample preparation:** 10/25/22
- **Date of gel:** 11/9/22
- **Date of WB:** 11/13/22
- **Sample:** RT4-D6P2T cell lysates
- **Washes:** 3x 3-min washes in Blotto
- **Last wash:** 1x Blotto, 2x TTBS
- **Exposure time:** 5 sec (NF-κB); 4 sec (Actin)
- **Corresponding figure:** Figure 2A

0 µg/mL LPS

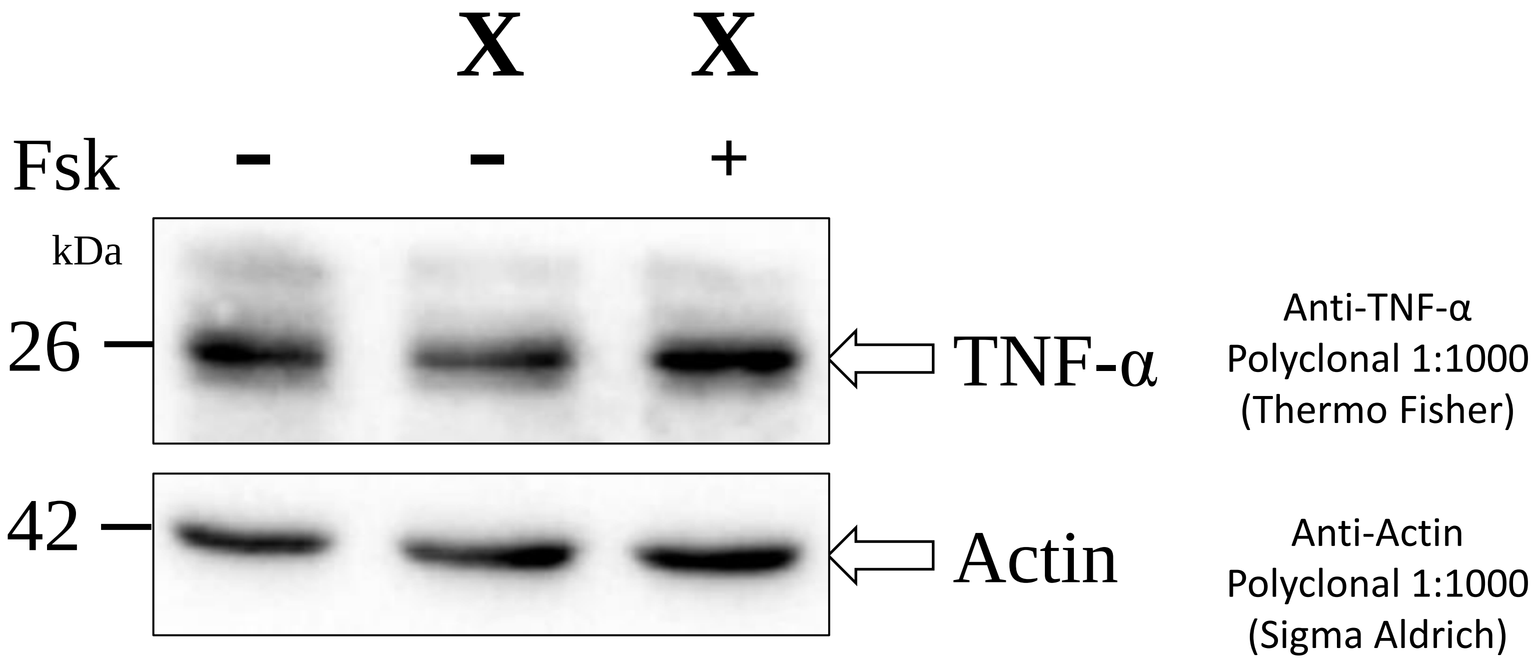

- **Date of sample preparation:** 10/25/22
- **Date of gel:** 11/9/22
- **Date of WB:** 11/13/22
- **Sample:** RT4-D6P2T cell lysates
- **Washes:** 3x 3-min washes in Blotto
- **Last wash:** 1x Blotto, 2x TTBS
- **Exposure time:** 15 sec (TNF-α); 6 sec (Actin)
- **Corresponding figure:** Figure 2B

0 μg/mL LPS

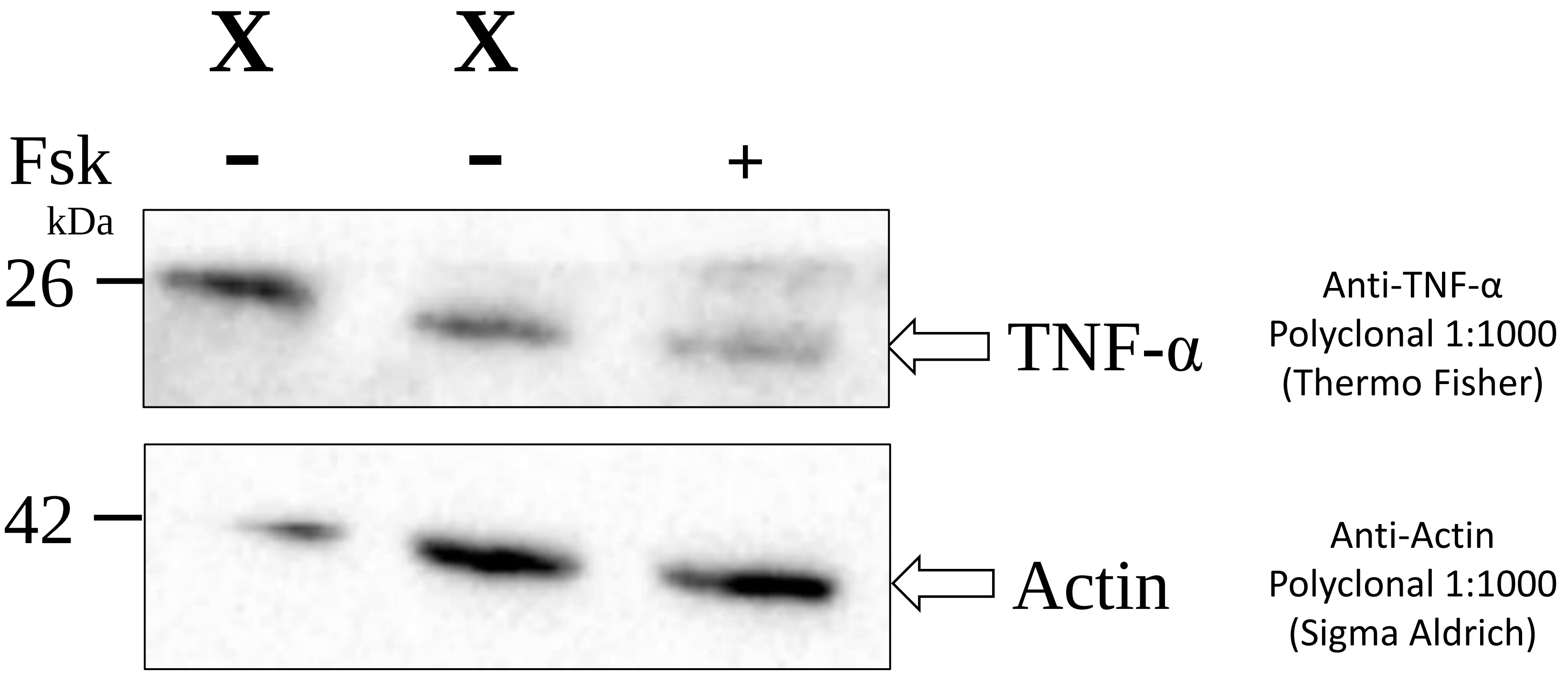

- **Date of sample preparation:** 9/27/22
- **Date of gel:** 10/4/22
- **Date of WB:** 10/11/22
- **Sample:** RT4-D6P2T cell lysates
- **Washes:** 3x 3-min washes in Blotto
- **Last wash:** 1x Blotto, 2x TTBS
- **Exposure time:** 100 sec (TNF-α); 10 sec (Actin)
- **Corresponding figure:** Figure 2B

0.1 µg/mL LPS

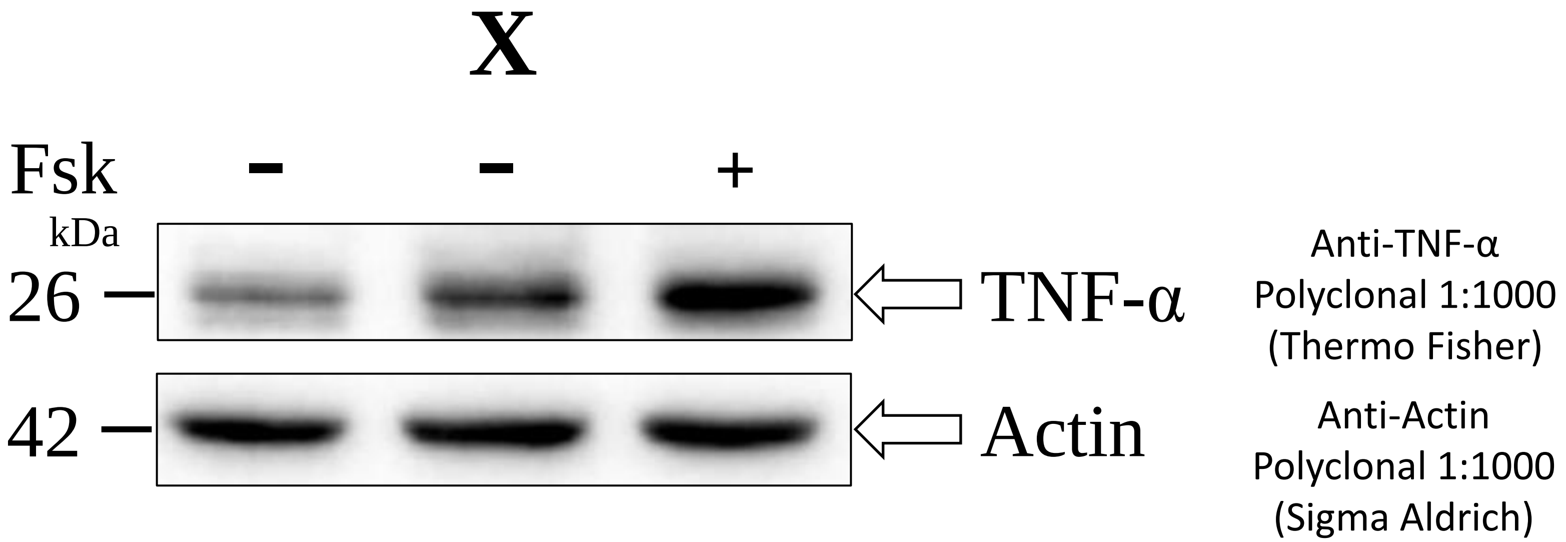

- **Date of sample preparation:** 10/25/22
- **Date of gel:** 11/9/22
- **Date of WB:** 11/13/22
- **Sample:** RT4-D6P2T cell lysates
- **Washes:** 3x 3-min washes in Blotto
- **Last wash:** 1x Blotto, 2x TTBS
- **Exposure time:** 10 sec (TNF-α); 4 sec (Actin)
- **Corresponding figure:** Figure 2B

1 µg/mL LPS

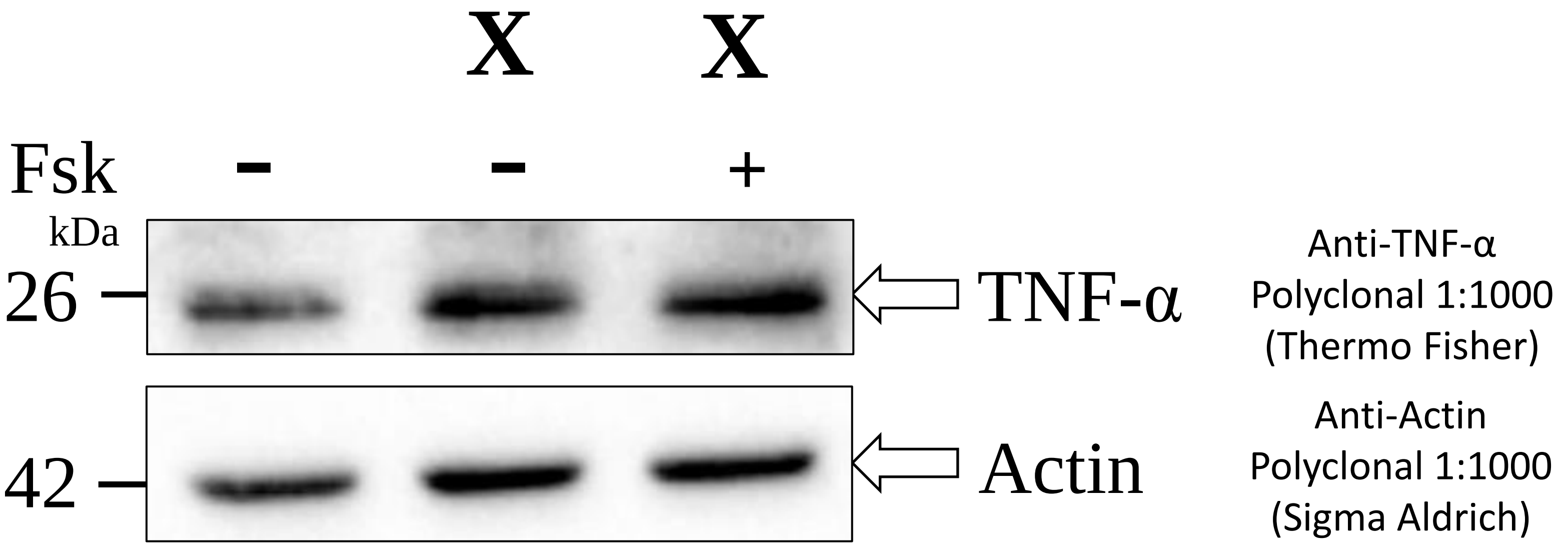

- **Date of sample preparation:** 9/18/22
- **Date of gel:** 9/20/22
- **Date of WB:** 9/21/22
- **Sample:** RT4-D6P2T cell lysates
- **Washes:** 3x 3-min washes in Blotto
- **Last wash:** 1x Blotto, 2x TTBS
- **Exposure time:** 100 sec (TNF-α); 3 sec (Actin)
- **Corresponding figure:** Figure 2B

1 μg/mL LPS

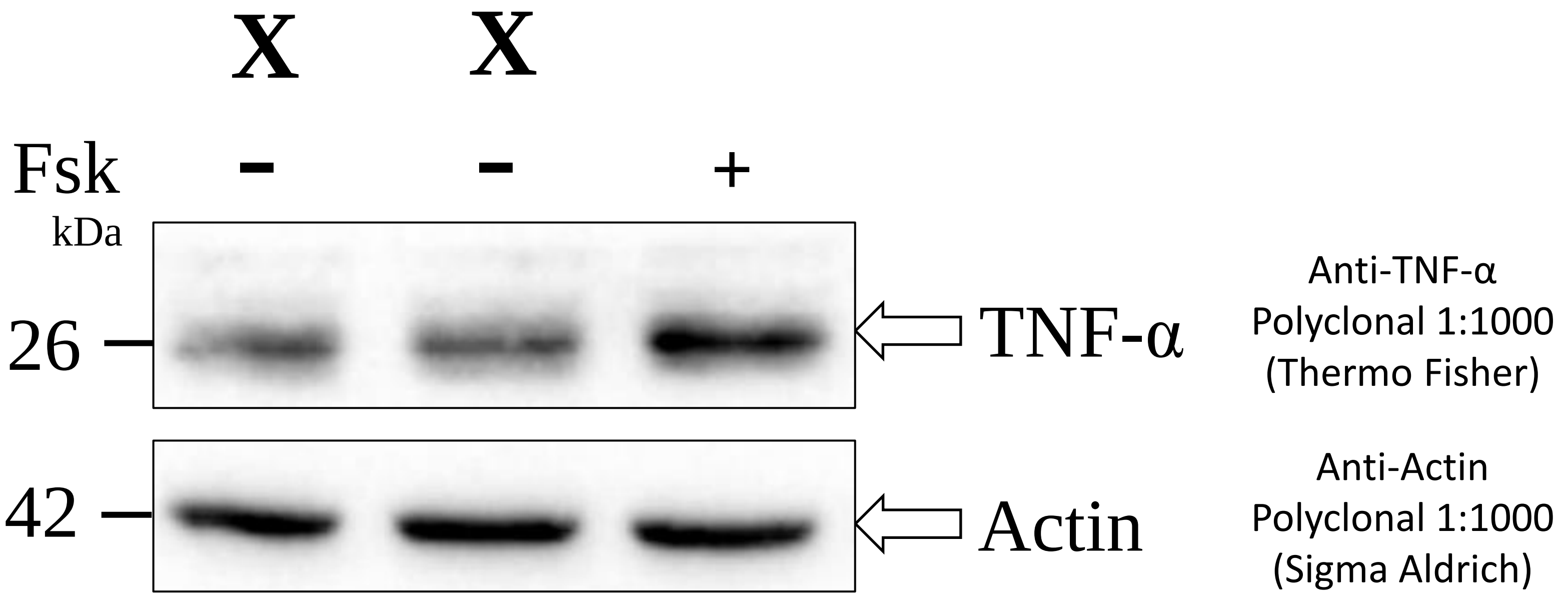

- **Date of sample preparation:** 10/25/22
- **Date of gel:** 11/9/22
- **Date of WB:** 11/13/22
- **Sample:** RT4-D6P2T cell lysates
- **Washes:** 3x 3-min washes in Blotto
- **Last wash:** 1x Blotto, 2x TTBS
- **Exposure time:** 15 sec (TNF-α); 5 sec (Actin)
- **Corresponding figure:** Figure 2B

10 µg/mL LPS

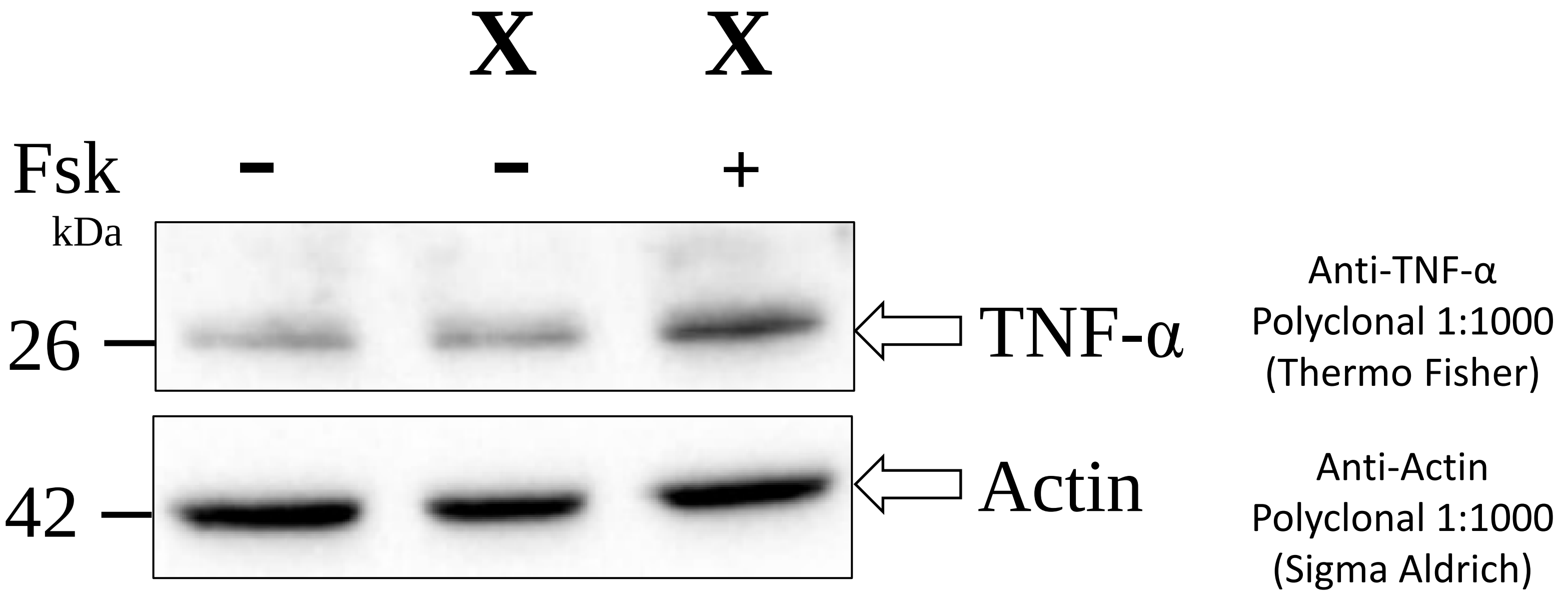

- **Date of sample preparation:** 9/18/22
- **Date of gel:** 9/20/22
- **Date of WB:** 9/21/22
- **Sample:** RT4-D6P2T cell lysates
- **Washes:** 3x 3-min washes in Blotto
- **Last wash:** 1x Blotto, 2x TTBS
- **Exposure time:** 100 sec (TNF-α); 4 sec (Actin)
- **Corresponding figure:** Figure 2B

10 µg/mL LPS

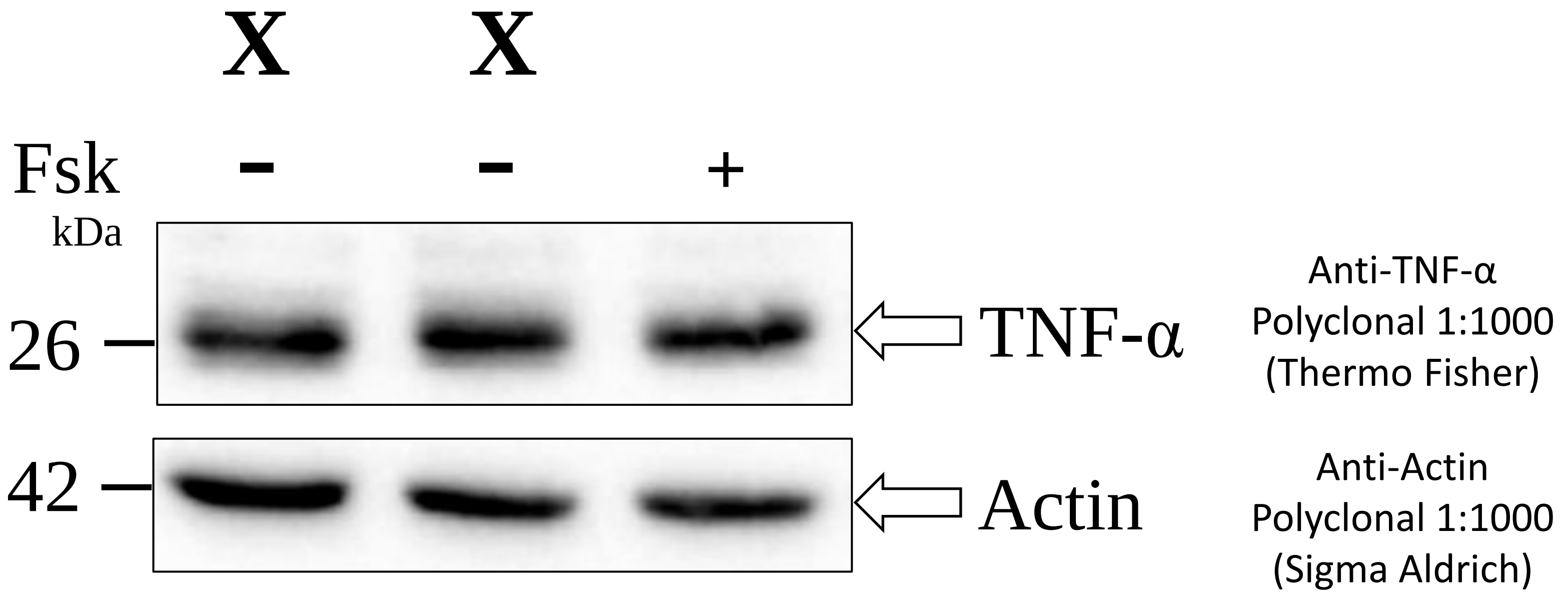

- **Date of sample preparation:** 10/25/22
- **Date of gel:** 11/9/22
- **Date of WB:** 11/13/22
- **Sample:** RT4-D6P2T cell lysates
- **Washes:** 3x 3-min washes in Blotto
- **Last wash:** 1x Blotto, 2x TTBS
- **Exposure time:** 25 sec (TNF-α); 4 sec (Actin)
- **Corresponding figure:** Figure 2B

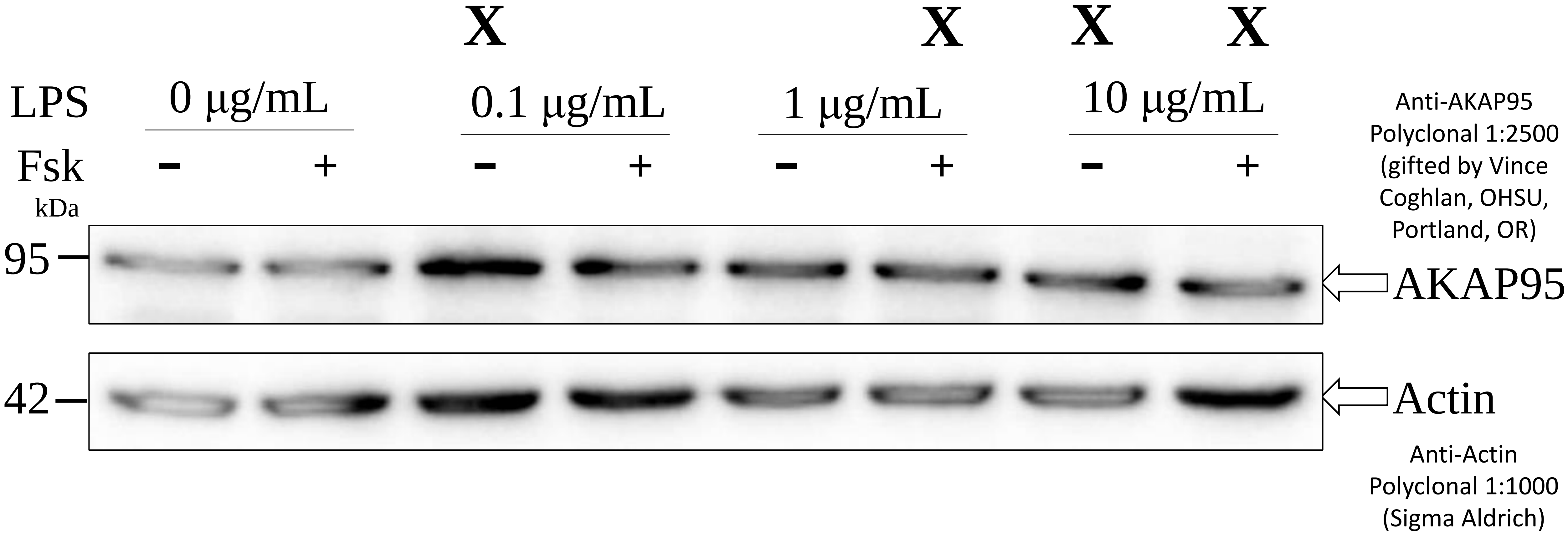

- **Date of sample preparation:** 2/2/23
- **Date of gel:** 2/7/23
- **Date of WB:** 2/9/23
- **Sample:** RT4-D6P2T cell lysates
- **Washes:** 5x 5-min washes in Blotto
- **Last wash:** 2x Blotto, 3x TTBS
- **Exposure time:** 1 sec (AKAP95); 1 sec (Actin)
- **Corresponding figure:** Figure 3A

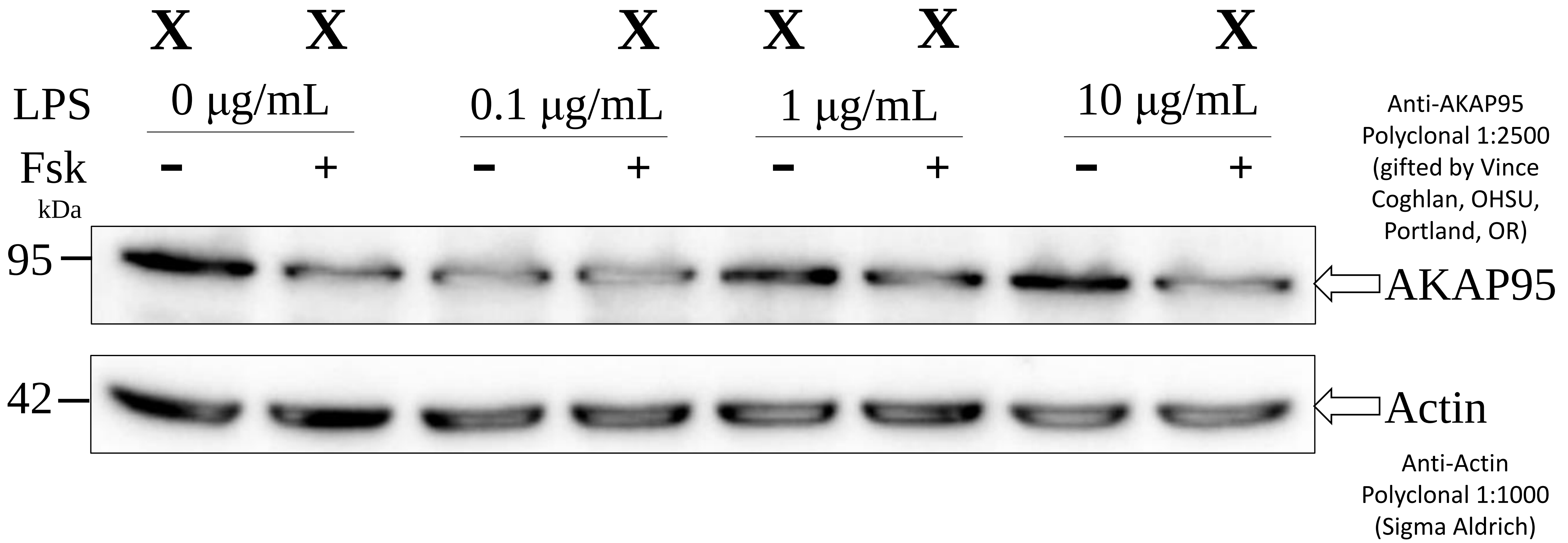

- **Date of sample preparation:** 2/2/23
- **Date of gel:** 2/7/23
- **Date of WB:** 2/9/23
- **Sample:** RT4-D6P2T cell lysates
- **Washes:** 5x 5-min washes in Blotto
- **Last wash:** 2x Blotto, 3x TTBS
- **Exposure time:** 1 sec (AKAP95); 1 sec (Actin)
- **Corresponding figure:** Figure 3A

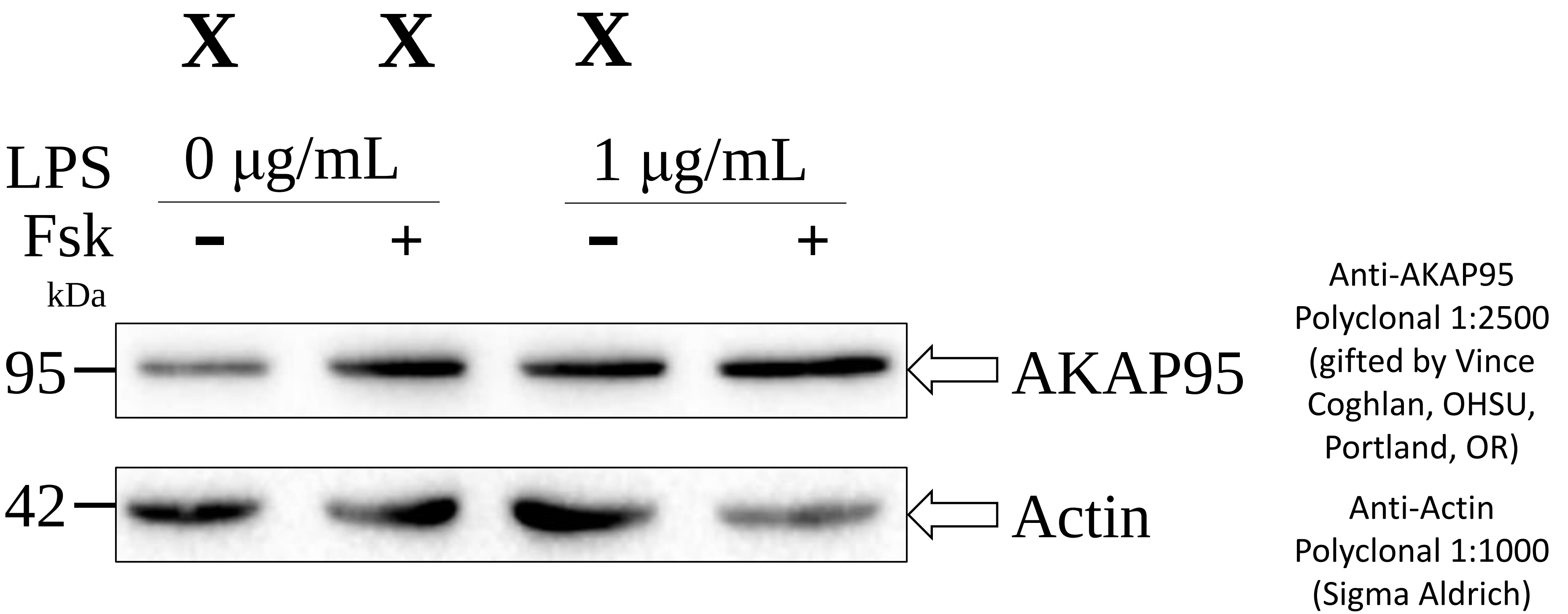

- **Date of sample preparation:** 10/25/23
- **Date of gel:** 1/27/23
- **Date of WB:** 1/29/23
- **Sample:** RT4-D6P2T cell lysates
- **Washes:** 5x 5-min washes in Blotto
- **Last wash:** 2x Blotto, 3x TTBS
- **Exposure time:** 2 sec (AKAP95); 10 sec (Actin)
- **Corresponding figure:** Figure 3A

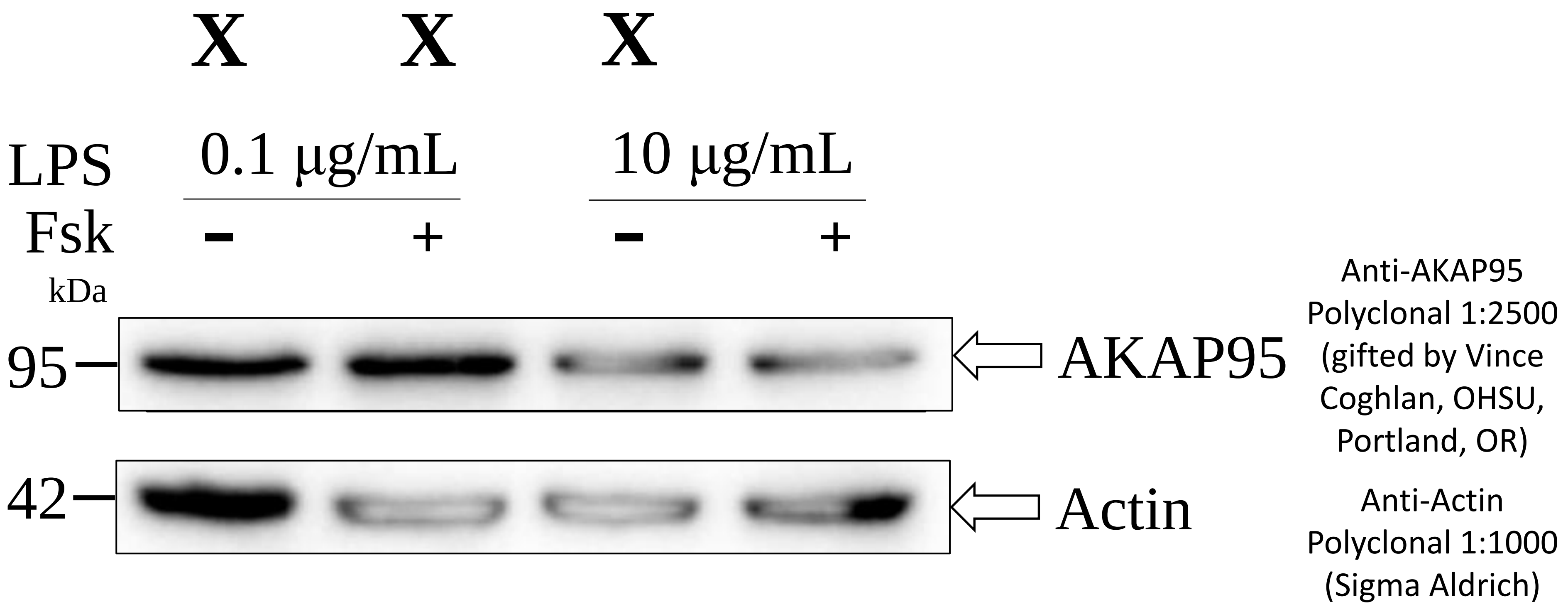

- **Date of sample preparation:** 2/2/23
- **Date of gel:** 2/7/23
- **Date of WB:** 2/9/23
- **Sample:** RT4-D6P2T cell lysates
- **Washes:** 5x 5-min washes in Blotto
- **Last wash:** 2x Blotto, 3x TTBS
- **Exposure time:** 1 sec (AKAP95); 1 sec (Actin)
- **Corresponding figure:** Figure 3A

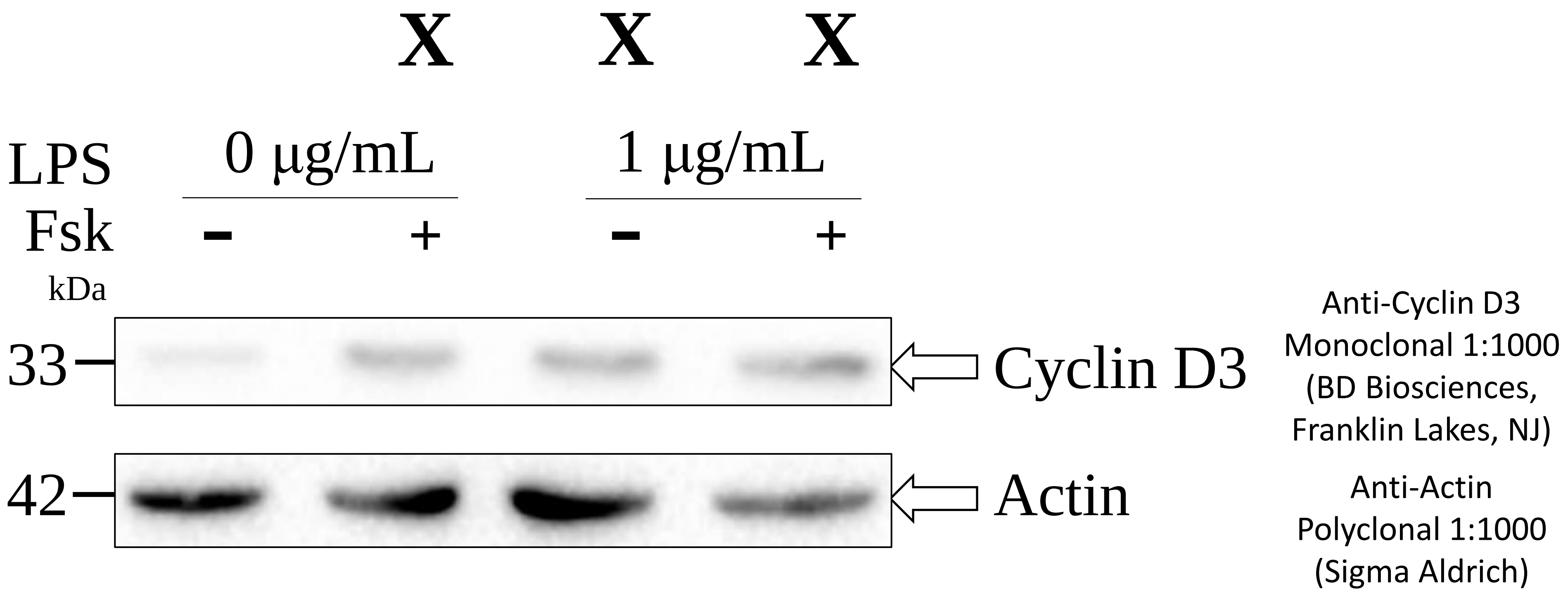

- **Date of sample preparation:** 10/25/23
- **Date of gel:** 1/27/23
- **Date of WB:** 2/13/23
- **Sample:** RT4-D6P2T cell lysates
- **Washes:** 5x 5-min washes in Blotto
- **Last wash:** 2x Blotto, 3x TTBS
- **Exposure time:** 60 sec (Cyclin D3); 10 sec (Actin)
- **Corresponding figure:** Figure 3B

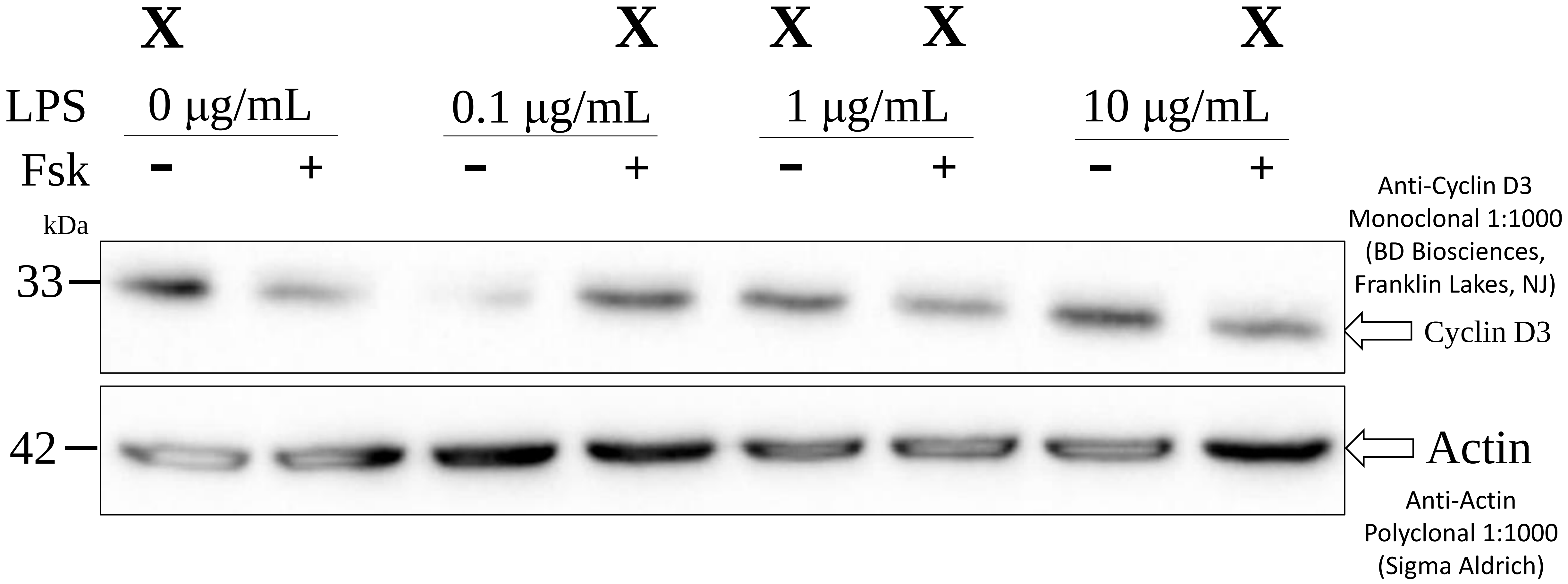

- **Date of sample preparation:** 2/2/23
- **Date of gel:** 2/7/23
- **Date of WB:** 2/13/23
- **Sample:** RT4-D6P2T cell lysates
- **Washes:** 5x 5-min washes in Blotto
- **Last wash:** 2x Blotto, 3x TTBS
- **Exposure time:** 43.3 sec (Cyclin D3); 1 sec (Actin)
- **Corresponding figure:** Figure 3B

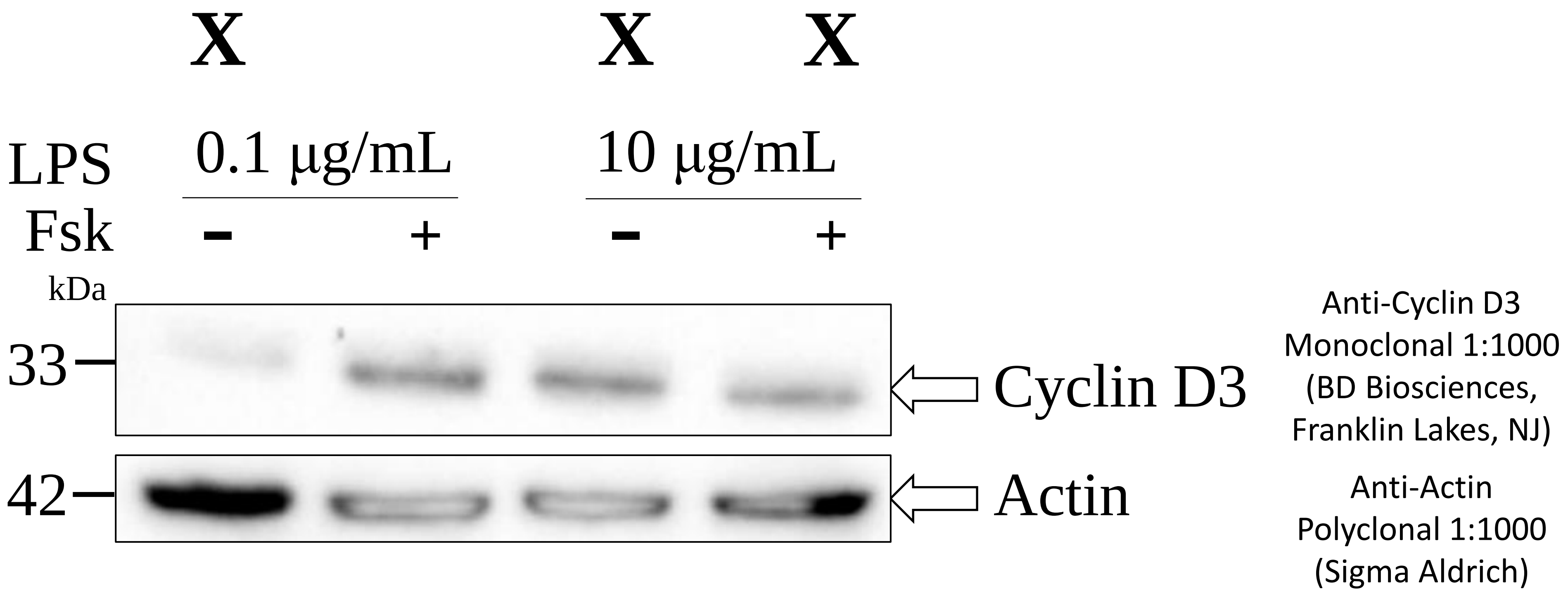

- **Date of sample preparation:** 10/25/23
- **Date of gel:** 1/27/23
- **Date of WB:** 2/13/23
- **Sample:** RT4-D6P2T cell lysates
- **Washes:** 5x 5-min washes in Blotto
- **Last wash:** 2x Blotto, 3x TTBS
- **Exposure time:** 60 sec (Cyclin D3); 1 sec (Actin)
- **Corresponding figure:** Figure 3B

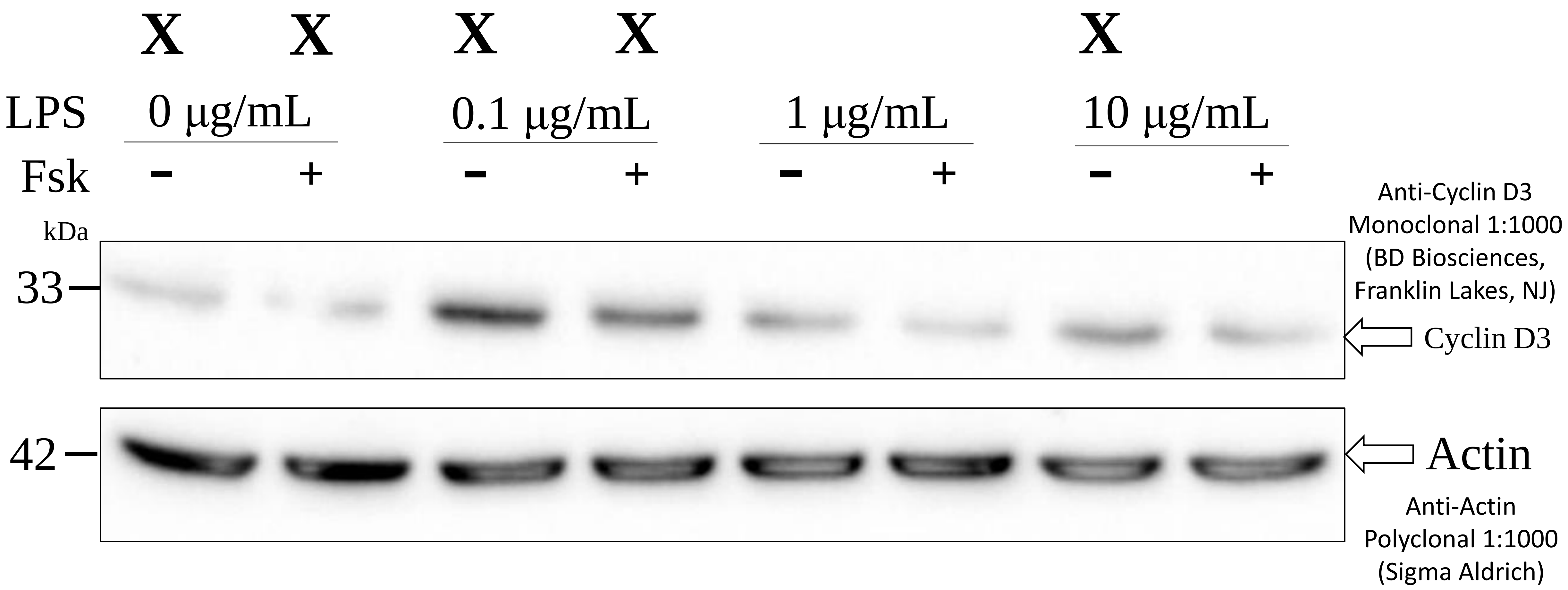

- **Date of sample preparation:** 2/2/23
- **Date of gel:** 2/7/23
- **Date of WB:** 2/13/23
- **Sample:** RT4-D6P2T cell lysates
- **Washes:** 5x 5-min washes in Blotto
- **Last wash:** 2x Blotto, 3x TTBS
- **Exposure time:** 48.9 sec (Cyclin D3); 1 sec (Actin)
- **Corresponding figure:** Figure 3B
